# Recent Issues and Configuration Factors in Perovskite-Silicon Tandem Solar Cells towards Large Scaling Production

**DOI:** 10.3390/nano11123186

**Published:** 2021-11-24

**Authors:** Mohammed Islam Elsmani, Noshin Fatima, Michael Paul A. Jallorina, Suhaila Sepeai, Mohd Sukor Su’ait, Norasikin Ahmad Ludin, Mohd Asri Mat Teridi, Kamaruzzaman Sopian, Mohd Adib Ibrahim

**Affiliations:** 1 Solar Energy Research Institute, Universiti Kebangsaan Malaysia, Bangi 43600, Malaysia; Noshinfatima1990@gmail.com (N.F.); suhailas@ukm.edu.my (S.S.); mohdsukor@ukm.edu.my (M.S.S.); sheekeen@ukm.edu.my (N.A.L.); asri@ukm.edu.my (M.A.M.T.); ksopian@ukm.edu.my (K.S.); 2 Information Device Science Laboratory, Nara Institute of Science and Technology (NAIST), 8916-5 Takayama-cho, Ikoma, Nara 630-0192, Japan; michael.jallorina.mf8@ms.naist.jp; 3 NAIST- École Polytechnique International Collaborative Laboratory, Nara Institute of Science and Technology (NAIST), 8916-5 Takayama-cho, Ikoma, Nara 630-0192, Japan

**Keywords:** perovskite-silicon, roll-to-roll, stability, solar cell, tandem

## Abstract

The unprecedented development of perovskite-silicon (PSC-Si) tandem solar cells in the last five years has been hindered by several challenges towards industrialization, which require further research. The combination of the low cost of perovskite and legacy silicon solar cells serve as primary drivers for PSC-Si tandem solar cell improvement. For the perovskite top-cell, the utmost concern reported in the literature is perovskite instability. Hence, proposed physical loss mechanisms for intrinsic and extrinsic instability as triggering mechanisms for hysteresis, ion segregation, and trap states, along with the latest proposed mitigation strategies in terms of stability engineering, are discussed. The silicon bottom cell, being a mature technology, is currently facing bottleneck challenges to achieve power conversion efficiencies (PCE) greater than 26.7%, which requires more understanding in the context of light management and passivation technologies. Finally, for large-scale industrialization of the PSC-Si tandem solar cell, the promising silicon wafer thinning, and large-scale film deposition technologies could cause a shift and align with a more affordable and flexible roll-to-roll PSC-Si technology. Therefore, this review aims to provide deliberate guidance on critical fundamental issues and configuration factors in current PSC-Si tandem technologies towards large-scale industrialization. to meet the 2031 PSC-Si Tandem road maps market target.

## 1. Introduction

The uncertainty in energy markets caused by the coronavirus disease-19 (COVID-19) pandemic has further highlighted the demand for renewable energy—Especially in the solar cell market—Due to the unreliability of oil as a constant source of income which may hinder the renewable energy resource market [1,2]. Accordingly, low-cost and highly efficient photovoltaic (PV) materials are immensely required as a source of renewable energy to overcome our dependency on fossil fuels in the context of minimizing global carbon footprint. Ideally, to economically compete with other clean energy resources, the cost of the PV module must be offset by highly efficient power output and operation cycle prolongation [3]. In theory, the need for tandem solar cells stems from the single-junction solar cell power conversion efficiency (PCE) intrinsic thermodynamics (Shockley-Queisser) limitation (i.e., around 29% in visible light range for the silicon) [4]. To date, the silicon heterojunction mono-crystalline silicon, inter-digitated back contact (IBC), and silicon heterojunction (SHJ) solar cells have shown sufficiently remarkable results on lab-scaled cell’s PCE > 26%. However, the state-of-the-art silicon solar cell technology is still struggling to offset around 3% from the calculated theoretical thermodynamic limit since 2017, limited by passivation and light losses [5,6]. Therefore, multi-junction photovoltaic or tandem solar cells (TSCs) represent a paradigm shift towards surpassing intrinsic single-cell limitations by stacking high bandgap solar cells over the bottom silicon solar cell [7].

In general, the multi-junction solar cell has proven its exceptional potential to produce PCE above 41%, with a theoretically expected PCE of 46% in the limit of no light concentration. Unfortunately, despite the improvement in PCE, the cost of PV modules is still high [8]. However, while targeting high PCE, tandem photovoltaic broad-spectrum research tends to be cost-oriented. The Levelized Cost of generated Electricity (LCOE) has been proposed to be significantly related to the long-life cycle of TSCs [9]. Consequently, in recent years, multi-junction (tandem) solar cells have enabled a relatively low-cost processing technique with superior efficiency than conventional homojunction and the rather expensive heterojunction solar cells [10].

Perovskite solar cells (PSCs) with intrinsic optoelectronic capabilities are projected to reach PCE over 29% by 2040 [11]. Research has shown that they can act as a top cell for silicon tandem cells due to their unique optoelectronic properties. Furthermore, economically added values of perovskite cells, such as ease of processing and relaxation of conventional lattice mismatch conditions, have enabled perovskites for further multi-junction processing [11]. 

In contrast with PSCs, silicon solar cells are technologically well-established due to their beyond visible light range bandgap (roughly from 500 nm to below 1200 nm), material abundancy, and proven microelectronic technology for a broad spectrum of research academia and industry [5,6,12]. Interestingly, the most outstanding record of the small-scale PSC-Si (~1 cm^2^) has attained PCEs of over 28% [13]. Nevertheless, the perovskite-silicon (PSC-Si) tandem’s detailed balance calculation reveals more than 40% efficiency that can be appropriately obtained at specific conditions [14]. However, the gap between theoretical and recent practical PCEs towards broad commercialization is still very challenging. Thus, even though the PSC-Si shows a promising upward trend, several intrinsic and extrinsic issues of the sub-cell and complete cell need to be addressed before the PSC-Si tandem could enter the solar cell market.

Though a 2021 International Technology Roadmap for Photovoltaic (ITRPV) projection [15] shows an outstanding Si-tandem solar cell market share, it is unclear whether perovskite solar cells may overcome a low lifetime issue (i.e., perovskite instability). Many interesting reviews on perovskite, silicon, and PSC-Si tandem solar cells have been published in the last five years, as illustrated in Table 1. However, whereas the focus on the perovskite has possessed many works of literature, the PSC-Si tandem solar cell with various configurational factors on each sub-cell detailed degradation and proposed mechanisms, and the prospect of the flexible substrate methods were less explored. Thus, in this comprehensive topical review on each sub-cell, we endeavoured the following:

- We present the current perovskite challenges and their possible solutions with a comprehensive concept of stability engineering. We provide our understanding and focus on the PSC intrinsic instability as the primary loss mechanism leading to multiple losses in device performance.
- We portray the prospects of PSC-Si tandem solar cells, focusing on the current champion PSC-Si solar cells’ most recent issues and obstacles.
- We explain the possible silicon solar cell technologies that may act as bottom cells in the PSC-Si tandem configuration, their advantages and disadvantages, market trends, and the shortcomings of providing wafer thinning requirements as possible routes for a cost-effective tandem based solar cell.
- The final section focused on the latest endeavours, challenges, and opportunities in roll-to-roll technology towards PSC-Si commercialization as a promising method of aligning thin-wafer silicon with PSC processing technology.

**Table 1:** Recent research gap studies on perovskite, silicon, and PSC-Si tandem solar cell.

Ref.no/Year	Author(s)	Focused Area(s)	Research Gap(s)
[16]/2016	Taesoo et al.	A broad review on thin-film solar cell technologies	perovskite detailed stability challenges, addressing methods, and PSC-Si tandem solar cell
[17]/2017	Kour et al.	A recent review on perovskite technology degradation mechanism and market challenges	perovskite ion migration challenges, addressing methods, and PSC-Si tandem solar cell
[11]/2018	Yamaguchi et al.	Topical review on all SHJ based tandem solar cell technology	Perovskite degradation mechanisms and various silicon solar cells structures
[18]/2018	Salhi et al.	Perovskite stability challenges understanding	Advanced PSC passivation methods such as two/three dimensional 2D/3D PSC and silicon-based tandem solar cell
[19]/2019	Krishnan et al.	Perovskite stability comprehensive review	Silicon bottom solar cell challenges and tackling methods
[20]/2019	Wang et al.	A comprehensive and detailed on perovskite stability	Hysteresis, halide free perovskite study, and PSC-Si tandem
[21]/2019	Yang et al.	Perovskite interface engineering	PSC additive/compositional engineering and PSC-Si tandem
[22]/2020	Hermle et al.	Passivating contacts for silicon solar cell in tandem configuration	Perovskite instability
[23]/2021	Akhli et al.	A detailed review on PSC-Si tandem with various configuration	Various silicon bottom cells technologies/roll-to-roll fabrication technology
[24]/2021	Wu et al.	Progress on PSC-Si tandem solar cell technologies	Various silicon bottom cells technologies/roll-to-roll fabrication technology
[25]/2021	Lui et al.	Detailed and recent progress on monolithic PSC-Si tandem	Four-terminal 4T PSC-Si tandem
[26]/2021	Kim et al.	Upscaling PSC-Si monolithic tandem strategies, including blading deposition	Perovskite loss mechanism and 4T PSC-Si tandem

## 2. PSC-Si Tandem Solar Cells Configurations

Researchers focus on innovative perovskite silicon tandem solar cells to achieve a highly efficient solar cell based on the proven and complex III-V elements-Si tandem solar cell technology [27,28]. Although III-V elements have provided adequate blue-shift light management, the considerable perplexity associated with III-V compounds solar cells, such as epitaxial lift-off deposition process and high material cost, has made researchers seek out cheaper and effective alternatives [28]. These alternatives are attractive because they are quickly processed and have direct band gaps, easily incorporated on a silicon sub-cell [29].

Technologically, three unique tandem cell structures are proposed by the research community; 2T (Two Terminals), 4T (Four Terminals) (Figure 1), and spectrally split tandem-based cell structures. Predominantly, while the spectrally split tandem cell is less cost-effective, this paper does not review spectrally split PSC-Si tandem cells due to the complex and expensive optics incurred in spectrally split PSC-Si even though it achieved high efficiency of beyond 28% [30,31]. In addition to the high cost incurred by the optical system, another concern is its unsuitability for adopting spectrally-split PSC-Si tandem due to light angular distribution sensitivity [32]. However, the standard classification is based on the choice of current contact terminals.

Generally, 2T tandem solar cells feature a straightforward structure with perovskite being monolithically deposited on silicon. Hence, it is primarily suitable for the existing production line integration. However, 2T current matching limitations require delicate sub-cell engineering. In contrast, 4T tandem solar cells relax the interconnection or recombination layer condition. The downside of 4T is mainly the extra electromagnetic spectrum loss due to separate window and interfacial layers (in the case of 4T) [33,34]. Furthermore, the 4T configuration dictates the economics of scale costs as the extra peripherals (e.g., wirings) would be reflected on module cost. Table 2 summarizes the most prominent PSC-Si tandem solar cells structures featuring the technical 2T and 4T advantages and limitations of 2T and 4T solar cells.

In comparison, perovskite-perovskite solar cell (PSC-PSC) and PSC-Si tandem solar cell have yielded over 24% PCE and 28% PCE, respectively, with the top absorber wide bandgap ~1.7 eV [9,13,44,45,46]. However, perovskite’s reliability issues and many complex phenomena impede its successful industrialization and wide implementation [47]. Hence, there is a compelling need for a parallel optimization process of top-cell perovskite while the entire tandem cell is being carefully designed. The upcoming sections of this work will portray the recent challenging issues of PSC-Si tandem and propose state-of-the-art solutions thematically.

Accompanying the upward trend of PSC-Si tandem cell efficiency, PCE has been boosted from 13.2% to over 29% in less than 10 years (irrespective of the terminal type of configuration). Furthermore, research efforts have been tailored to the economic feasibility of material and processing costs, making potential tandem cells’ future market more viable over single-junction solar cells [48,49,50,51,52]. As a result, in 2020 and 2018, with various cell sizes, Helmholtz-berlin and Oxford announced a world record of 29.15% and 28% certified perovskite-silicon tandem, respectively, followed by IMEC-Belgium with 27% and EPFL- Switzerland (25.3%) [40,50,51,52]. A summary of the prominent PSC-Si tandem solar cells metrics and limitation are briefly detailed in Table 2.

**Figure 2:**
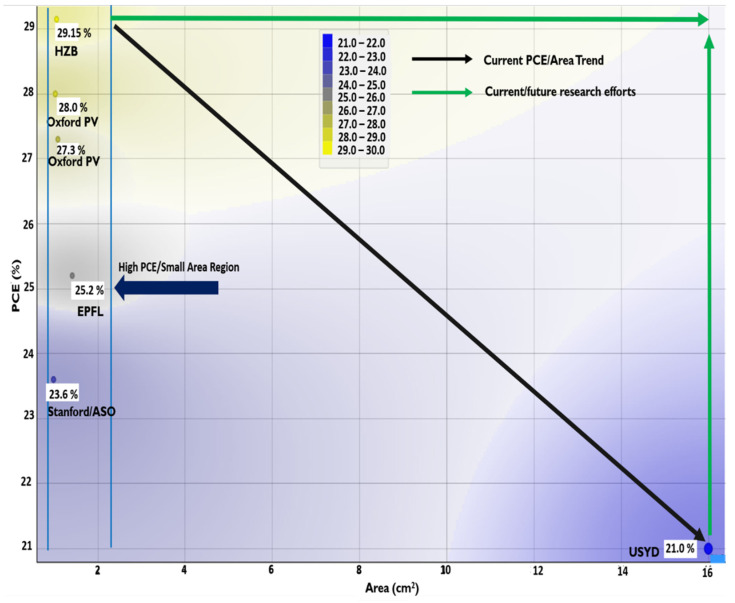
PCE (%) as a function of the area in cm^2^ for certified PSC-Si tandem solar cell for various structures since 2016–2020 shown in the navy-blue color zone margins. The lowest right coroner represents uncertified 2018 released by University of Sydney (USYD)-Australia [64]. The figure shows current PCE-Area and research/future trends in the colored arrows. Data has been extracted from solar cell efficiency tables (49–57) available at www.onlinelibrary.wiley.com.

Another recent success with 26.3% PCE has been achieved by Italian research groups, which overcame the technological process complexity of the previously produced monolithic 2T multi-junction technologies via 4T transformation based on the mechanical bonding technique [53]. A PCE of 25.7% was recently revealed through the direct solution process using thick perovskite film achieved under a collaboration between KAUST and the University of Toronto [54]. In addition, PSC 2D-coated 3D (integrated low dimensionality) perovskite showed better stability and passivated surface with 27.7% PCE and 26.2% IBC and PERL silicon, respectively [55]. Considering the silicon solar cell recorded the highest PCE of 26.7%, the perovskite silicon tandem can reach around 45% theoretical limit [56,57]. Meanwhile, this technical advancement of PCE in a brief period by highly dedicated research groups has been certified. As in Figure 2, the perovskite silicon multi-junction solar cell market’s success is obstructed by sub-cell and the composite integrated tandem cell issues. In Figure 2, the size of the presented tandem cell is limited by the size of the perovskite top cell. The inverse correlation between PCE and the active area is so far attributed to perovskite’s low fill factor as a result of high series resistance and various recombination mechanisms [58], which requires careful engineering using new materials, interfaces engineering, and sheet resistivity reduction techniques. The perovskite cell’s stability is another challenging problem that will be detailed in the next sections.

## 3. Perovskite Solar Cell as Top Cell

Given the progress timeline during 2020, Perovskite solar cell efficiency reached >25% in a decade, superseding silicon solar cells for a similar time [59]. Thanks to the perovskite intrinsic magnificent optoelectronic properties, easy technological process, and high availability of materials. Furthermore, Perovskite optical response is corroborated by high visible light absorption utilization of up to 70% of the incident photons [60], making perovskite an effective absorber compared to inorganic silicon-based active layers [61]. Moreover, perovskite carrier diffusion length in the order of 1µm is higher than silicon by a notable difference, which is reportedly due to the bulk near crystalline low defect level concentration [62,63].

### 3.1. Perovskite Solar Cell Structure

Diverse structures have been tentatively proposed for perovskite photovoltaic. Classifications are based on various layers of chemical composition involved in the physical generation of photo carriers (active layer) and other buffer layers. Buffer layers vary for electron transport layer (ETL) and hole transport layer (HTL), which ideally should pass through the optical light/generated carriers to/from the active layer [17]. Electronically, a buffer layer is commonly introduced to induce a proper energetic band bending through band matching with both absorber/active layer and metal contacts [65]. Ideally, perovskite solar cells are implemented in various configurations; typical or regular/standard (n-i-p) and inverted (p-i-n) structures. Schematic illustrations of PSC structures can be found elsewhere [66,67].

Commonly, the standard chemical structure of the perovskite active layer with no derivatives satisfies ABX_3_, where A site typically hosts organic/inorganic elements(s). In contrast, the B and X sites are filled with inorganic metal and halide elements, respectively [68]. Thus, perovskite photovoltaics’ performance and structural stability properties are profoundly influenced by each chemical site composition, precursor, solvent, and crystallization engineering strategy. A conventional well-cited fundamental structure is tetrahedral organic-inorganic mixed-halide methylammonium lead iodide (CH_3_NH_3_PbI_3_ or MAPbI_3_) perovskite, which has received wide attention due to its superior optoelectronic properties [36,69,70]. However, the structure for perovskite absorbers does not have to stick to the basic MAPbI_3_ because highly efficient perovskite cells involve absorbers with mixed cationic (multi-cationic) chemical compounds, as in Table 3. Interestingly, ever since the first reported perovskite solar cell, researchers have been trying to establish the merits of uniquely designed solar cells. However, since many perovskite constituent layer’s materials and intrinsic physiochemistry properties vary enormously, the search’s starting point was the geometrical evaluation of perovskite bulk using the Goldschmidt (*t*) criterion in EQ 1.

(1) $$t=\frac{r_{A}+r_{O}}{\sqrt{2}\left(r_{B}+r_{O}\right)}$$

where *r_O_* is the radius of oxygen; *r_A_* and *r_B_* are the ionic radii of the *A* and *B* cations, respectively.

The Goldschmidt tolerance factor (*t*) represents the first formalism of perovskite stability based on the distinctive ionic radii geometrical size of the ABX_3_ perovskite constituents. However, the uncertainty associated with tolerance factors as a design tool will be discussed in the figure of merits limitations section [71].

**Table 3:** List of examples of efficiency performance-related material types and perovskite absorber chemical structures.

Material (Compound)	Perovskite Absorber	PCE (%)	Ref.
Organic	(FAPbI_3_)_0.95_ (MAPbBr_3_)_0.05_	22.7 ± 0.51	[72]
Organic	(FAPbI_3_)_1−x_(MAPbBr_3_)_x_	21.6	[73]
Organic-Inorganic	FA0_.75_(MA_0.6_Cs_0.4_)_0.25_PbI_2_Br/Rb(5%)	17.4	[41]
Organic-Inorganic	PTABr-CsPbI_3_	17.06	[74]
Organic-Inorganic	Cs_x_FA_1−x_Pb(I,Br)_3_	14.0	[39]

Upscaling of perovskite solar cells is profoundly required for successful industrialization, utilizing the advantages of optoelectronic properties and inexpensive cell processing methods [58]. However, since 2009, many challenges have demanded in-depth investigations for innovative, diverse materials, structures, and technological methods [16,58,68], increasing the selection process’s complexity. 

### 3.2. Implementations and Challenges of PSC

While tandem photovoltaic devices represent an essential block in overcoming single-junction (homo-junction) limitations, PSC-Si tandem economic feasibility is well studied [75]. Recently, Zhengshan J. et al. [38] performed a detailed balance techno-economic study on tandem solar cells. With high certainty, the study revealed a feasibility condition on a module cost level basis. It has been proven that the tandem cell would lose a market advantage if the top cell were more expensive than the bottom sub-cell. Therefore, the relative module cost to the balance of the system (BoS) cost ratio favors the cheap top sub-cell compared to the bottom sub-cell. Thus, the main concern for the realization and large-scale industrialization of PSC-Si tandem configuration is perovskite’s durability which may hamper the optimistic techno-economic studies outcomes.

Furthermore, hysteresis, surface defects, and ionic segregation involve materials’ eco-suitability, thus limiting technology upscaling [76,77]. Each of these loss mechanisms will be highlighted in the following sub-sections on a priority basis. However, all loss mechanisms of perovskite top cell implementation will be treated strategically and cumulatively. In other words, a comprehensive study on the device stability loss mechanism (next) is essential to promote PSC-Si tandem cells, which is lacking in the literature. Therefore, standardized reports on PSC solar cell is highly needed to understand the physics underlying loss mechanisms.

#### 3.2.1. Perovskite Solar Cell Stability

A typical state-of-the-art perovskite with the highest PCE is currently being demonstrated on a small scale in the order of ~0.1 cm^2^, as presented in Figure 3a,b. Therefore, PSC small size can be significantly observed from the inverse relationships between perovskite’s PCE and area due to low fill factor (FF) and solution method (i.e., spin coating) limited large cell size, as in Figure 3a (inset) [19,58,78,79,80]. Typically, the other main factor that hinders perovskite from vast market presence is its inherent scaling instability. However, the ongoing research progress is still working on this challenge. R. Wang et al. in Ref. [20] elaborated on the various proposed instability mechanisms associated with perovskite geometrical instability factor metrics. They also discussed the pathways of perovskite loss factors. This work distinguishes instability into intrinsic instability from extrinsic losses as both effects influence the mainstream PSC-Si tandem solar cells.

##### A. Perovskite Intrinsic Stability

Remarkably, research has already established a link between perovskite instability and every material constituent, such as intrinsic and extrinsic materials’ properties and interactions, electronic states, doping, deposition methods, and device structures [18,19]. Arguably, the inherited (intrinsic) effect could have resulted from the ion migration effect [35]. Even though the perovskite may be highly encapsulated, associated factors due to poor intrinsic instability exist. Due to its volatility, the irreversible chemical reaction between bulk perovskite constituents poses a challenge in the trade-off between perovskite efficiency and stability. Hence, we speculate PSC intrinsic instability likely to be the initiator for the domino effects for the entire device instability. So far, the most efficient perovskite solar cell involves locally formed isolated phases PbI_2_ and CH_3_NH_3_I due to the intrinsic chemical reaction, which requires particular attention in addressing this challenge [20]. Hence, we may infer that inherent compositional engineering plays a vital role in perovskite instability. Recently, Zhang et al., in their timely review of perovskite stability, showed that there is inconsistency in the thermodynamic evaluation methods results at room temperature [80]. Hence, perovskite intrinsic requires more understanding of the degree of enthalpy or entropy on the given perovskite ionic components and thermodynamic data quantification availability for a given precursor with enough informative materials database. The uncertainty in perovskite materials’ intrinsic thermodynamic instability may undermine other encapsulation strategies to be adopted. Therefore, the understanding of perovskite constituents’ thermodynamics is critical for stability design rules. For instance, a clear understanding of the PSC-HTL intrinsic interface has driven PSC-Si tandem PCE up to 29.15% [35]. Hence, detailed knowledge of physiochemistry and charge extraction rate is required to address perovskite instability issues.

Intrinsic stability is also a structurally dependent function; for example, mesoporous-based electrodes such as TiO_2_ have shown better stability than planar electrode-based perovskite [20]. This may drive further research on the notion of the superiority of nanostructures incorporated in perovskite device engineering. Furthermore, the chemical interaction between perovskite’s various constituent layers is another intrinsically challenging issue. The acidic PEDOT:PSS was shown to intrinsically react with transparent conductive oxide (TCO) in addition to its hygroscopic nature, which may drive perovskite research with the promise of alternative stable HTL materials and HTL-free layers [82,83]. Moreover, the metallic iodide formation due to the interaction between perovskite carrier layers and the metallic contact could be considered as perovskite instability intrinsic pathway [84,85]. Hence, interfacial engineering with pinhole-free and 2D materials is highly required to address perovskite intrinsic stability issues.

##### B. Perovskite Extrinsic Stability

The extrinsic instability of perovskite with the introduction of environmental stimuli such as moisture, oxygen, and light negatively affect PSC-Si tandem solar cells. Aside from the intrinsic reliability of perovskite, the extrinsic effects are discussed in the literature with the underlying proposed degradation mechanisms and proposed solutions in Refs. [19,20].

Moisture is always discussed as the most detrimental factor for perovskite solar cell stability as an extrinsic agent. The Amine group is considered the primary degradation pathway due to the affinity between a water molecule and hydrogen, producing a hydrated product [19,80]. Hence, sulfur and/or compositional engineering would effectively address perovskite moisture instability [86]. Furthermore, 2D/3D perovskite has also proved to be promising grain boundaries passivation and moisture blocking methods due to the 2D perovskite exhibiting good hydrophobicity [87]. However, 2D perovskite carrier transport anisotropy could be strategically researched as a perovskite-enabled capping or encapsulation supporting method.

Degradation of perovskite materials has been researched for the influence of environmental oxygen on structure decomposition. So far, the synergetic effects of oxygen and light degradation have shown that the formation of superoxide as a result of the light-induced photoelectron is the possible cause for oxygen formation [88,89]. Therefore, the search for perovskite materials with less oxygen reactivity and high binding energy could address oxygen degradation [89,90]. On the other hand, ultraviolet (UV) light has been considered another extrinsic perovskite degradation mechanism. Proper interfacial engineering with barrier layers carrier transport has shown to be an effective method to tackle the UV light effects on perovskite [91,92]. However, UV light degradation requires specific engineering as the perovskite top cell is critical for blue-shift light spectrum absorption.

Remarkably, 2D perovskite and compositional engineering were proven as effective methods to address perovskite thermal stress mechanisms. However, thermal stability is of paramount importance for perovskite stability as the standard operating conditions require a continuous temperature of 85 °C for a specific time [87,93,94].

Many promising approaches have been proposed to maximize device stability in recent research compiled in Table 4 [83,84] to address the short lifetime of perovskite cells. Table 4 shows the interplay between the extrinsic and intrinsic proposed physical degradation mechanisms and their best corresponding mitigation/elimination strategies, the chemical decomposition interplay between the original perovskite structure constituents may initiate unstable solar cells [20]. To the best of our knowledge, there are many proposed solutions based on the current comprehensive understanding corresponding to each stability degradation mechanism.

##### C. Stability Figure of Merits Evaluation

As explained earlier, the geometrical factor maps the tolerance factor to the ideal cubic perovskite structures [123]. However, one may question the validity of this method as a structural stability evaluation tool for the broad spectrum of perovskite materials. Even though the Goldschmidt criterion assessed the stability of various perovskite structures (Table 3) as well as the extendibility of double perovskite structure (AB_2_X_6_), its accuracy is often insufficient for all perovskite functional materials [124,125]. Moreover, more than one Goldschmidt tolerance factor makes it a confusing perovskite stability evaluation tool [71]. Christopher et al. showed a revised version of the Goldschmidt tolerance factor having an extendibility of almost 92% of the materials, which may overcome the generic Goldschmidt tolerance factor predictability limitations [126].

Therefore, even though stability-based geometrical tools such as Goldschmidt represent an empirical or semi-empirical evaluation tool, structural stability is still considered a heuristic tool for the general design of perovskite due to the limitations in understanding the various intrinsic and extrinsic agents’ interaction with structural stability. For instance, under the ionicity different size assumption, Formamidinium (FA^+^) HC(NH_2_)_2_^+^ ion has a better tolerance factor ratio t ~0.99 under thermal stress than the methylammonium CH_3_ NH_3_^+^ ion. However, despite the superiority of FA over MA ion in resisting extrinsic effects such as light and thermal stress, FA^+^ shows low reliability in the presence of moisture, which implies shortcomings in stability-based geometrical assessment methods as a fundamental tool [123]. Hence, there is a need for a more comprehensive figure of merits based on the physical understanding of overall perovskite instability. Furthermore, the relationship between perovskite constituents’ ionic radii size and lattice constant hypothesizes a stability trade-off for the corresponding extrinsic agents as experiments show good stability.

Meanwhile, the stability response is weak for the same ionic radii; it requires delicate tuning through perovskite compositional engineering. To this end, a standardized and comprehensive geometrical design tool is needed to facilitate upfront engineering, such that the stability predictability becomes more certain sensitive to the new materials inclusions. Furthermore, schematically, the major interrelated extrinsic and intrinsic factors that place obstacles in developing perovskite solar cells can be conceptualized in Figure 4.

As explained earlier, we may sum up the perovskite instability factors in Figure 4a, which portrays the entire perovskite degradation mechanisms manifested in extrinsic and intrinsic effects. The humidity, thermal stress, and UV light are the main external factors of the perovskite solar cell, which requires extensive perovskite encapsulation engineering research, as depicted in Figure 4b. Although extrinsic effects are considered a triggering means for the perovskite decomposition, the intrinsic instability factors in Table 4, such as active layer low activation energy of decomposition, carriers transport layers, and metal electrode reaction, are likely to be the leading cause for the ion segregation mechanisms, free surface states and hence hysteresis. Therefore, we can understand that intrinsic perovskite instability is the primary area driving mechanism on which the perovskite research shall concentrate. We have observed that most perovskite research may focus on several engineering areas in Figure 4b. However, collective and comprehensive engineering (herein, we refer to as stability engineering) is highly required to address perovskite intrinsic stability. In light of Table 4 various solution methods for the PSC instabilities, we propose the concept of stability engineering as a guiding and formal design tool rather than handling one specific engineering method and neglecting the other design methods.

#### 3.2.2. Perovskite Hysteresis Effect

Predominantly, even though the latest state-of-the-art PSC-Si tandem had experienced almost negligible hysteresis due to better composition engineering of perovskite, long-term hysteresis scenarios have yet to be investigated. Hysteresis signifies the looping behavior of the parametric function for various systems [127,128]. In technological process terminology, Hysteresis indicates system instability and loss of controllability [129]. Similarly, current density-voltage mismatch sweeping (J-V) behavior is considered an unwanted aspect of future photo-efficient perovskite solar cell improvements [130,131]. Zhenggi et al. showed that there is an unconfirmed hypothesis on the origin of hysteresis. However, to a certain extent, hysteresis is attributed to the intrinsic reversible Ferro-polarization of perovskite materials, ion migration, and contact charge trapping or contact polarization (see Figure 4a) [131,132]. Another understanding of the hysteresis effect can be thought of initially due to intrinsic perovskite materials stability. The intrinsic decomposed perovskite materials such as PbI_2_ induced ion segregation (see next section), and hence interfacial defects induce a hysteresis effect in PSC. Lin et al. [87] proposed that perovskite low dimensionality can hinder hysteresis through high crystallinity degree control. In that sense, 2D/3D stacking structures demonstrated the hysteresis elimination method and defects and ion migration healing method [87]. Recently, the high passivation of trap density has been proven as an effective potential method for hysteresis suppression [133,134,135]. However, based on the research as mentioned earlier and PSC-Si tandem solar cell, we may suggest that PSC solar cell passivation strategies need to be transformed to the tandem level utilizing low dimensionality materials such as 2D, so it may assist in acquiring stable and negligible/hysteresis free PSC-Si tandem solar cell.

#### 3.2.3. Perovskite Ion Segregation

Perovskite ion segregation is a critical factor in PSC-Si tandem as it is closely related to hysteresis and perovskite top cell instability. Therefore, it is of the utmost importance to look into PSC-Si’s ion segregation effect. One of the perovskite’s detrimental factors is ion migration (i.e., charged defects) under bias voltage or thermal drift, as displayed in Figure 4a [136]. In a typical perovskite reference structure, it is evident that MA ion concentration is higher compared to iodide ion, yet iodide ion (I^+^) diffusivity is more elevated in magnitude than MA^+^ [137]. However, the primary reasoning is debatable. Lee and co-workers [136] quantified that the intrinsic reasons for ion migration were high defective perovskite and low-temperature solution preparation method (i.e., thermal instability). However, other researchers proposed that ion segregation may be caused by precursor solution ionicity and the propensity of perovskite compounds/elements having a weak bond (activation energy). Anderes et al. concluded that there is relevance between ion segregation and increased grain boundaries with iodide-rich domains, which indicates the direct correlation between grain size and ion segregation [138]. Hence, it is suggested that composition and morphology properties play a vital role in understanding and suppressing ion migration effects [137,138,139,140,141]. Therefore, enhanced crystallinity suggests uniformed crystal orientation will decrease grain boundaries. However, an exception for a high bandgap halide-rich perovskite can be beneficial for performance, representing a trade-off compromise for top cell perovskite in tandem configuration. It was found that mixing halide bromine (Br) and iodine (I) in the light induces compositional segregation due to Pb: Halide excess stoichiometry [139]. Further information on optimum halide ratio versus bandgap can be found in Ref. [58].

Moreover, ion segregation reportedly decreased due to higher lateral size than smaller ones in the bulk perovskite [140]. Nevertheless, as mentioned in the earlier literature and the conflict raised in the data provided for ion segregation, the agreement on A and B cations alloying with improved crystallinity could be a possible solution for the light-induced anion phase ion segregation [141]. A recent study showed that a minimum intensity threshold of 90 °C is enough to induce ion segregation with thermodynamic analysis [142]. Furthermore, a. Mahapatra et al. demonstrated with additive engineering for various interfacial materials that the enhancement was not only in terms of efficiency, but hysteresis, surface, and bulk states were almost eliminated or diminished [143]. However, perovskite-sustained elemental and/or compound thermodynamic data are crucial for understanding and tackling ion segregation mechanisms/pathways. Furthermore, we promote perovskite high crystal along low dimensional (2D) research as the ultimate research path towards ion- segregated and hysteresis-free perovskite.

#### 3.2.4. Perovskite Free Surface States

The role of surface states in the PSC-Si tandem was not intensively investigated. However, surface states near the band edges are definitively considered a detrimental factor for J-V characteristics due to the low charge extraction rate [144]. The effect of surface states typically leads to an undesirable point, which is intrinsic Fermi level pinning (FLP) in GaAs semiconductors and organic semiconductors [145,146]. In organometallic halide perovskite, the effect of Fermi pinning takes place at the interface due to surface state density [147]. The impact of FLP is critical to metal contact and perovskite charge transport layer band alignment. Therefore, getting an accurate perovskite FLP measurement is another cumbersome to be well studied [147]. Thibaut et al. developed an accurate FLP physical characterization method, which can be considered a standardized characterization protocol [148]. In addition, it is suggested that the fluctuations of the electrical properties in polycrystalline materials arise due to grain-to-grain variations and not due to distinctive electronic properties of the grain boundaries [148,149,150]. Since the conclusive origin of FLP in perovskite is still under debate, many methods are being considered to understand FLP conditions and reduce surface recombination. Lately, quad-cationic engineering with strontium addition has shown great healing of interface states [151].

Furthermore, it was shown that surface morphology improved for C_60_/perovskite due to the addition of strontium [151]. Another study of interest involves interface engineering, which requires more investigation into perovskite’s defective nickel oxide (NiO_x_)HTL layer. As a result, NiO_x_ created an ohmic contact due to NiO_x_’s tendency to fix the FLP close to the valence band maxima [152]. Therefore, we think work function (WF) engineering is critical to mitigating FLP in PSC. Further improvement in the stability context through interface engineering strategies can be found in Ref. [21]. Furthermore, interface engineering demonstrated a proper pathway towards the highly efficient champion PSC-Si tandem solar cell (PCE 29.15%) through HTL optimization [35]. Given that the ambiguity lies in high quality and repeatable perovskite film process conditions, we believe that Al-Ashouri et al. [35] study is one of the most promising methods to address intrinsic interface recombination, which can be extended to the metal contact surface recombination mitigation.

#### 3.2.5. Perovskite Eco-Toxicity

Although this issue can be considered of minor importance in PSC-Si tandem solar cells, lead toxicity remains of paramount concern for current and potential perovskite manufacturers. Kim et al. [77] discussed that a large perovskite module has to pass through less hazardous chemical routes for PSC fabrication [77]. However, an overall understanding of the toxic materials/elements spill rate is lacking for accurate toxicological impact studies [153]. On the one hand, a lead amount in PSC that might be considered extremely harmful lacks a quantification study). On the other hand, lead-free perovskite is known to experience facile oxidation in the presence of air, which poses a challenge for the research community to innovate more suitable alternatives for Pb (lead-free perovskite).

Moreover, to the best of our knowledge, laws, and regulations have not yet been established specifying the permissible amount of lead to be used in future perovskite solar cell processes. Even though many techno-environmental studies on several PSC-Si tandems have been conducted [154], perovskite’s stability failure mode needs to be further investigated to keep silicon bottom cells operable and potentially reduce perovskite material’s environmental impact during perovskite top cell failure. We believe another exciting point that is interesting for lead-based perovskite solar cells is materials recycling/reuse. Recently, Chen et al. [155] showed that more than 99% of the Pbl_2_ can be reused with weakly acidic cation exchange resin from the decommissioned perovskite solar cells. However, we think recycling economic barriers such as proper and standardized recycling technologies are yet to be attractive for recycling agencies and companies. In other words, low-cost solar cell recycling requires research and financial incentive to be widely accepted for various perovskite solar cell recycling treatments.

## 4. Silicon Solar Cell as Bottom Cell

Silicon is an abundant and non-toxic material with proven microelectronic applications for more than 50 years [156]. With above 29% estimated thermodynamic theoretical efficiency, the current state-of-the-art silicon solar cell technology is close to reaching the theoretical limit of attaining a stable PCE of 26.7% [6,157,158]. However, one solution is abridging the expected single-cell limitation via multi-junction (i.e., tandem) solar cell technologies [159,160,161]. By and large, looking into current PSC-Si bottom cell, the challenging aspect of silicon photovoltaics lies (with the exclusion of current mismatching) in producing highly efficient solar cells with low capital expenditures (CapEx), O&M, and hence low module cost that may compete with other electrical power sources [162,163,164]. Technologically, this can be achieved by adopting low-cost, highly passivated development of thin single/heterojunction quality cells (advanced new concept cell technologies), which is conceived as a probable limitation of silicon state of the art solar cell for tandem application. A review of the state of art silicon bottom solar cells potentially applicable to PSC-Si tandem solar cells is portrayed in the next section.

### 4.1. Silicon Solar Cell Performance Metrics

**Figure 5:**
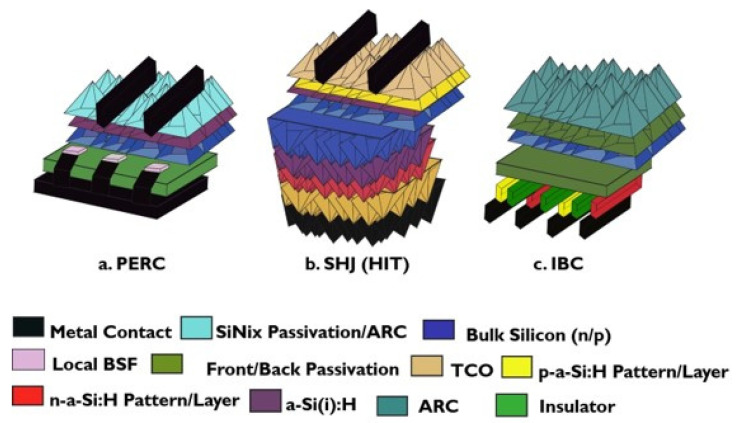
Generic and fragmented silicon solar cell technologies cross-section schematics (The surface morphology may vary from flat surface/partially textured to the fully textured surface) From Left to right (**a**) PERC technology, middle (**b**) SHJ technology and (**c**) IBC. All structures and materials are for an explanatory purpose reprinted from [186], Copyright (2016), with permission from Elsevier (Not to Scale). (Note: in PERC structure, back metal contact in the black color is intended to diffuse through insulator via local BSF layer).

Since the inception of the first homojunction silicon solar cell in the 1950s, the main motive for developing highly efficient solar cells has been cost reduction [165,166]. Hence, numerous research and advancements have been carried out on silicon types such as polycrystalline, mono-crystalline, and amorphous silicon [167]. While deposition, diffusion, and characterization technologies are undergoing remarkable development in the passivated emitter and rear cell (PERC) solar cell Figure 5a, the focus on heterojunction solar cells intensified. In silicon heterojunction solar cells (SHJ), which constitute most of PSC-Si bottom solar cells, the use of pure mono-crystalline silicon absorber in conjunction with high-quality passivation led to various forms of built-in and selective surface electrical fields [168,169]. Figure 5 shows several silicon solar cell technologies via cross-section schematics, which indicates the different structures, passivation layers, and materials. On the other hand, even though IBC technology Figure 5c has been demonstrated in 3-Terminal PSC-Si tandem solar cells [170], the mainstream research objective for highly efficient laboratory-based heterojunction crystalline silicon solar cell (SHJ), as in Figure 5b, is benefiting from SHJ structural symmetry. Therefore, the requirements for a highly efficient new concept of silicon bottom solar cells are:

- High-quality purified and thin absorber (i.e., kerf loss reduction) [171,172,173];
- Introduction of high-quality surface passivation with better light transmission and electronically tunneling layers of polycrystalline silicon on oxide (POLO) technology and its derivative technologies such as tunnel oxide passivated contact (TOPCon) harmonized with PERC technology forming PERx/TOPCON/(PERC+) solar cells, which may benefit from existing PERC production facilities [174,175,176,177]. The most recent SHJ solar cell research on passivated contacts investigates a shift of local selective contact from very thin a-Si: H to a new concept of self-doped and high/low work function (hole/electron) adapted materials. Examples of high/low work functions are transition metal oxides (TMOs) optimized for the front and rear contacts [178,179,180,181,182];
- Adoption of advanced and cost-effective new concepts for light trapping techniques to sustain wafer thinning technologies [183,184]; and
- The high-quality metallic contact layer is optimized using fewer materials and more industrial high throughput production of metallic contact [174,185].

### 4.2. Implementation and Challenges of Silicon Solar Cell

Recent studies established four criteria of silicon cell-based device loss according to each parameter-dependent cell efficiency. The four criteria are carrier recombination analysis, optical analysis, resistive loss, and cost reduction [187]. Table 5 shows the main performance metrics, overall loss mechanism(s), and technology–commercial gap for the silicon solar cell technologies depicted in Figure 5.

Silicon heterojunction solar cell (SHJ) sub-cell technology offers a promising future in PSC-Si tandem cell bottom cells [43]. A high-quality SHJ absorber features high-quality passivation of symmetric structure with an enhanced open-circuit voltage close to 750 mV [181,188,189,190]. Hence, more focus on present silicon solar cell challenges will emerge from this technology. Despite SHJ-based technologies having less recombination loss in comparison with widely industrialized technologies such as passivated emitter and rear cell (PERC), as shown in Figure 5a, and aluminum-back surface field (Al-BSF) [174,176,177], SHJ technology encounters optical loss due to the intrinsic and doped hydrogenated amorphous silicon and TCO bilayers [191].

As for parasitic optical loss analysis, high/low work function transition metal oxide compound for hole and electron promises to play a role in reducing intrinsic and doped a-Si:(H) parasitic layer losses [192,193]. Concerning reflection loss, back-contacted SHJ with double Anti Reflective Coating (DARC) has been proven to be an effective way to reduce optical reflection current [194,195]. On the other hand, Cruz et al. proposed the TCO’s couple pathway optimization routes through thin TCOs that complement optical thickness of ARC and a new competitive candidate replacement of the conventional ITO [196]. Lately, compounds such as aluminum-doped zinc oxide (AZO) have shown the potential to replace scarce ITO components for more than 22% PCE [195]. For prospects, 2D materials such as graphene are currently optimized to yield over 97% transmittance. However, graphene possesses high sheet resistance in comparison with ITO. One possible solution for high sheet resistance of graphene is through chemical and/or hetero-doping [197]. More research on this area is required to attain the ultimate trade-off between light transparency and low resistivity.

Resistive loss presents an extensive challenge, limiting solar cell performance, clearly manifested in fill factor parameter value. Fill factor (FF) is a corresponding parameter highly influenced by recombination current, series, and shunt resistance. Hence, for proper characterization, it is necessary to decouple the influence of each FF-dependent parameter [194,198,199]. As for series resistance, a trade-off with novel materials is required to outweigh each SHJ bilayer conductivity and passivation function [200]. It is imperative to have good TCO coverage as it is necessary to avoid an edge effect. Due to TCO’s edge effect, the low shunt resistance may lead to high leakage current [201,202].

Finally, silver (Ag) metallization and wafer process cost concern the module level because they are considered significant costing challenges [173,203]. While high series resistance metal fingers aspect ratio and paste conductivity must be balanced, especially in thin silicon wafers, silver metallization cost can be offset by optimized screen-printing and copper plating deposition. Furthermore, TCOs can be considered as a potential replacement for the expensive Ag [22]. Therefore, an updated and systematic techno-economic study represents a promising way through the highly efficient thin wafers for overcoming module cost ($/kW) and CapEx ($/kW/Yr) alongside maintaining a plateau Levelized Cost of Electricity (LOCE) ($/kWh) curve. Therefore, frequent and updated techno-commercial studies are crucial for assessing the wafer thinning approach as a promising route for cost reduction, which requires optimization of slicing technologies through loss reduction (kerf loss) and contact materials optimizations [171,173].

**Table 5:** The overall performance metrics for a bottom cell selection criteria, loss mechanism (s), and leading silicon technologies market perspective.

Technology	Performance Metrics	Loss Mechanism(s)	Market Share State/Anticipation
PERC	- less Surface Recombination Velocity (SRV) in comparison with Al-BSF [171,187]	- Bulk, emitter, and back contact SRH recombination [187] - Front and back reflector optical loss - High Al consumption [204]	- Current mainstream silicon along Al-BSF photovoltaic. - Anticipated shift from p-PERC to n-PERC reaching 70% total solar cells market share by 2022 then fading away for PERx/TOPCON/PERC+ [205].
PERC+	- Less SRV in comparison with PERC with better passivation technologies - Less Al paste consumption in comparison with PERC [171,204]	- Slight increase in series resistance in comparison with PERC [204]	- The market share was expected at 16% by 2019 [204] - A 2021 recent projection PERC+ to be the leading market stream in the upcoming years [15] - -Anticipated to be the leading horse for the tandem applications
SHJ	- Better passivation quality as compared to homojunction and IBC-SHJ cell (Figure 5b) [6] - Less thermal budget used in SHJ [181]	- Front and back contact layers optical loss [181] - Balancing between TCO’S optical and electrical series resistance performance [181] - Effect of the edge recombination due to TCO’S inadequate coverage [143].	- International Technology Roadmap for Photovoltaic (ITRPV) expects SHJ to possess a 12% market share by 2026, a more significant share than 15% by 2031 [15,206]
IBC-SHJ	- Better optical response due to no emitter contact shadowing (Figure 5c) [181] - Comparable V_oc_ and FF to SHJ [6] - A promising new paradigm with IBC POLO [174]	- Process complexity (i.e., lithography) and cost are the main concerns for this technology [181]	- ITRPV market share expectation at 15–20% by 2030 [15,207]

## 5. Fundamental Issues and Configuration Factors in PSC-Si Tandem

Regardless of the various PSC-Si tandem configurations, the PSC-Si tandem solar cell is essentially influenced by each sub-cell loss mechanism separately elaborated in the previous sections of this article. Additionally, composite tandem layers possess a portion of the entire tandem cell loss mechanisms, which require more investigation in current and future research. As stated in the perovskite section, instability is the primary concern for perovskite to be widely industrialized, affecting the whole tandem derivatives industrialization. Hence, from all previously produced perovskite that showed cell instability, a common observation is that the aperture size was within the range of 1 cm^2^. For the sake of large-scale industrialization, there is a need to research positive zone paths (in green color), in Figure 2. Despite efforts of tandem lifetime prolongation through various technologies such as 2D/3D-based perovskite, the aperture size is below industrialization requirements [54,208]. Therefore, perovskite instability, ion movement, and hysteresis must be understood before fully commercializing PSC-Si tandem solar cells in the context of stability engineering.

In addition to the perovskite instability, one fundamental monolithic multi-junction photovoltaic requirement is each sub-cell current matching (band matching) [57]. However, even though the perovskite absorber enables band tunability characteristics, technologically, top cell in tandem configuration suffers from reaching the optimum bandgap with high-quality and thick films in the order of 1 µm [209,210,211]. It has been reported that perovskite film thickness and morphology control are constituent parts for better performance; hence the need for more market-oriented and controllable deposition technologies can be proposed as an active research area [211,212,213]. Another degradation mechanism is carrier selective contact reactivity, as elucidated by K. Bush et al. [42].

One of the scalable process selection criteria is process compatibility within PSC-Si absorbers and sub-cell complementary layers [102,211]. Consequently, research focuses on increasing inorganic-based perovskite’s PCE and low thermal budget layer, thanks to the maturity of inorganic-based chemistry and the invention of novel deposition methods, such as photonic curing methods, which need to be developed to be upscaled for large PSC-Si tandem solar cells.

As for the bottom cell, the route for PSC-Si tandem cell has been well established with the most promising silicon solar bottom cell technology [76,107,214]. In most cases, the mainstream technology undertaken for PSC-Si tandem has been established with SHJ bottom cell technology, as in Table 3. However, SHJ’s first challenge has been explained earlier in terms of optical and carrier recombination loss. Another disadvantage of SHJ that has caused a delay in its introduction to the market is its higher cost than the PERC cell [215].

In addition to the parasitic absorption loss exhibited in the entire PSC-Si tandem cell, incompatibility process conditions between top and bottom tandem-based cell integration pose a material selection challenge. However, besides the perovskite sequential deposition technique by authors in Ref. [39], Lamanna et al. [53] produced an innovative breakthrough in overcoming the technological process for monolithic perovskite silicon solar cells. Their method was achieved by decoupling various process temperature conditions of top cell perovskite from the bottom silicon, where: (a-Si:(H) layer is designed only to tolerate temperatures below 200 °C. Moreover, this unique independence feature has allowed for direct mechanical bonding over the randomly textured silicon surface while relaxing perovskite surface roughness conditions [53].

Similarly, a promising method to overcome the challenges of solution-based perovskite deposition on roughly textured silicon surface was covered by Yi Hou et al. It was carried out by depositing an increased molar ratio perovskite solution to the top of the silicon, which yielded a cumulative cell efficiency of 25.7% [54].

**Figure 6:**
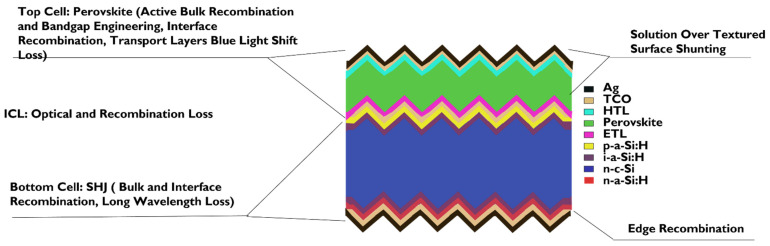
An illustration of the Generic 2T perovskite silicon tandem major loss mechanisms with each set of layers light spectrum absorbance adapted from various literature (The surface morphology may vary from flat surface/partially textured to the fully textured surface). The purple circles represent the modified ICL layer (i.e., nanocrystalline materials). [Reprinted/Adapted] with permission from [ref [219]] © The Optical Society.

**Figure 1:**
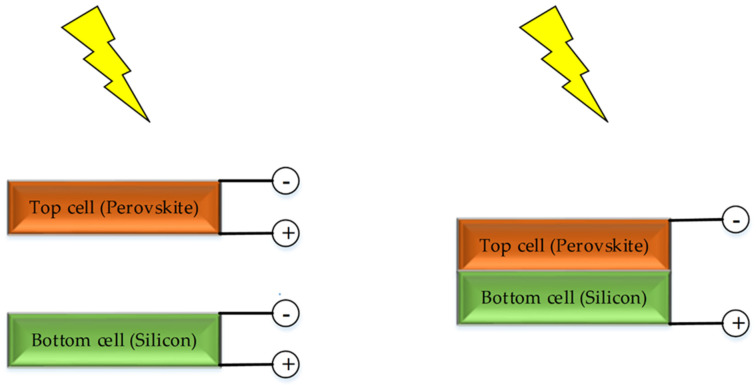
PSC-Si 4T (**left**) and 2T (**right**) simple schematic diagram.

**Figure 3:**
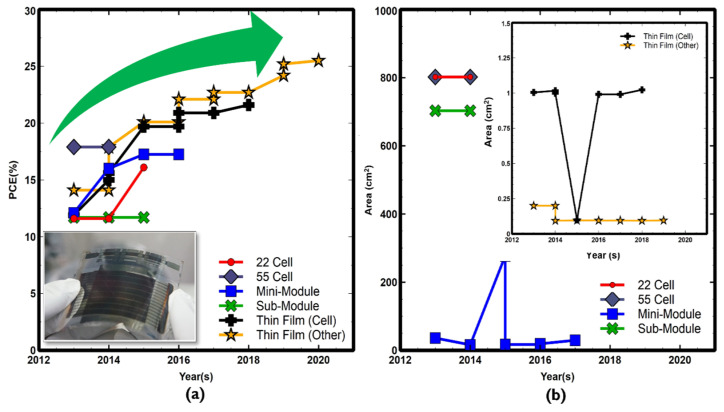
Illustration of the certified various perovskite cell and multi-cells, (**a**) the efficiency trend, the inset shows real minimodules produced by Toshiba [81]; (**b**) the area of the corresponding cells for the same period for higher cells area, and the inset represents the corresponding thin film (various) small area graph. Some data points in Figure 3 coincide with each other. Data are obtained from solar cell efficiency tables (42–57) available at www.onlinelibrary.wiley.com.

This new 2T concept needs to account for the intermediate recombination or tunneling layer (ICL) design (Figure 6). A nanotechnology-based ICL nanocrystalline structure has been revolutionized and demonstrated in the subsequent PSC-Si tandem solar cell, cited in Table 2 [37,38,216]. In addition, an organic recombination layer with other materials has been proposed for low carrier recombination, high light transparency, and as a protective layer for subsequent bottom cells [27].

Even though industrial PSC-Si tandem cells may require more research to realize feasible lossless ICL on a large-scale basis, scientists have improved the optics in the 2T tandem through a high surface-to-volume ratio of nanostructured (nanocrystalline silicon) recombination layer [38,216,217]. Typically, nano-based structure applications are not just limited to ICL applications. For instance, the requirement for transparent perovskite using a silver nanowire electrode has vastly extended and dramatically improved light transparency even though perovskite instability might occur from the chemical interaction between silver and segregated halide.

However, in the next section, perovskite band tunability and silicon J_sc_ and FF are discussed. Furthermore, Figure 6 schematically summed up all open research challenges for the 2T monolithic PSC-Si tandem.

### 5.1. Band Gap Tunability in Perovskite Sub-Cell

Recognition of the high open-circuit voltage obtained in perovskite solar cells is due to the apparent good passivation. On the contrary, as explained in the previous section, tuning the high/low bandgap is not a trivial task due to expected variability in the high throughput production line (see Figure 6). Therefore, ongoing research adopts methods and technologies that have enabled tunable perovskite bandgap through advancement in materials, processes, and triple tandem cell-based technology.

At present, many examples of novel bandgap engineering in perovskite have been attained and become more pronounced through compositional engineering and technological process advancements [34,216,218]. For example, Sahli et al. exploited the sequential deposition process alongside compositional halide engineering for tuning bromine/iodine ratio content for a relatively stable perovskite. Their structure demonstrated a perovskite bandgap of 1.63 eV through cationic engineering using inorganic-organic Cs-FA mutations. However, the obtained perovskite bandgap was far from the optimum top cell bandgap of 1.75 eV [216]. Perovskite bandgap tuning has obtained 1.94 eV with the conventional solvent technique and improved crystallinity utilizing potassium additive, contributing to the suppressed ionic migration and hysteresis effect reduction [10]. Remarkably, Werener et al. applied the triple junction PV with open device circuits of 2.7 V by adopting 1.8 eV and 1.4 eV for optimized perovskite top cells and middle cells, respectively. However, the drawback of the optimized triple cell bandgap is that FF had to be sacrificed due to the current mismatching limitation raised from the bottom cell [39]. Hence, the optimized high bandgap of perovskite is not adequate for obtaining high voltage and FF values. Instead, high and non-variable compositional engineering is required to obtain high open-circuit voltage in the top cell.

### 5.2. Jsc and FF Evaluation in Silicon Sub-Cell

Low J_sc_ is not a significant concern for the four-terminal (4T) PSC-Si tandem solar cell as each sub-cell is mechanically stacked on each other. However, repetitively stacked solar cell bilayers in separate sub-cells increases module-related costs, such as wiring cost. While the two-terminal 2T tandem is more straightforward in process and cost than the 4T, each cell’s current matching represents significant limitations. These limitations are due to current mismatching in the bottom-cell bandgap [217]. Steve et al. showed that the low Jsc limited low temperature monolithic PSC-Si tandem with 18% efficiency due to the cell’s flat surface [43]. The adoption of symmetric surface texturing as light trapping management along with indium-doped zinc oxide (IZO) trans-conductive layer in the tandem block has shown a modest improvement in J_sc_ [52]. A very close PCE of 25% at 1.4 mA cm^−2^ was demonstrated by Luana e. al. with an adequate back textured surface and intermediate refractive index matching layer [217]. However, in the 2T PSC-Si tandem cell configurations, the optimum trade-off between the cumulative short circuit current and open-circuit voltage needs more optimization without sacrificing FF with delicate design and less production variability.

In all PSC-Si tandem bottom cells, FF was shown to be drastically reduced due to the texturing of perovskite on the silicon surface, which in turn undermined the bottom cell’s FF [216]. Furthermore, Werner et al. observed that front surface texturing did not influence free carrier absorption at 550–800 nm; instead, FF was influenced by perovskite solution-based deposition on the textured surface, leading to the undesirable lateral carrier transports and shunt resistance reduction [32]. On the other hand, with less FF cell loss, perovskite co-evaporated conformal deposition methods over textured silicon surface suit the low-scale tandem, which is not feasible for large-scale PSC-Si tandem solar cells. Therefore, the technological process for perovskite conformal coating needs further research towards market scale cell size.

## 6. Future Perspectives towards Large-Scale Industrialization

By looking into uncertain present energy conditions due to COVID-19, the solar energy market needs to compete with conventional energy resources. However, existing silicon technologies such as the current mainstream market for solar cells are set for anticipated cost reduction schemes. Therefore, this review aims at providing some future perspectives on the large-scale solar cell industrialization, whereas PSC-Si cell mating may attract investors’ attention in the upcoming periods. Our proposed industrialization roadmap supports other roadmaps suggested by academia in adopting metal electrodes and simple reduced functional layers [220]. However, the suggested roadmap emphasized perovskite stability engineering, thin silicon advanced passivation understanding, current matching improvement in 2T PSC-Si, and the exploitation of ongoing roll-to-roll perovskite solar cell technology. Furthermore, iterative testing protocols are updated to match the existing IEC- based reliable silicon testing methods.

Even though silicon possesses the highest PV market share and attracts many anticipated efforts on PSC-Si industrialization from 2018 [221], the research society argues that silicon market share is not up to the commercial 2030 baseline target. A struggling PCE of 26.7% evidence this since 2017 [6]. As such, the realization of high-quality highly-thin silicon solar cells (~<50 µm) requires a revolution of wafer passivation, slicing process, and kerf loss reduction technologies (Figure 7) [171,222], which requires critical research of passivation characterization techniques. Moreover, we recommend the review of Rehman et al.’s study [223] for passivation prospects in line with current technologies.

Technological process challenge in cost reduction through high production line effective throughput is also required for further feasible industrialization of PSC-Si. For example, Hasse et al. showed that upscaling, laboratory-scaled low-pressure chemical vapor deposition (LPCVD), and plasma-enhanced chemical vapor deposition (PECVD) downsides need to be overcome on a technological basis process to accommodate commercially passivated contact processes. Furthermore, thermal vapor deposition metallization requires more research on finding feasible and effective upscaled alternatives [22]. On the other hand, the 200 °C metallization is not a limitation for the silicon bottom cell, but the top cell, where perovskite top layers cannot tolerate this temperature. Hence, the low process and high throughput metallization gap need more top cell adaptation research [224]. Therefore, under perovskite top cell low process temperature, we expect the breakthrough in the POLO-based silicon solar cell [175], which recently reached a PCE~26.1%, may take place only in the upcoming 4T tandem cell benefiting from an existing production line.

**Figure 4:**
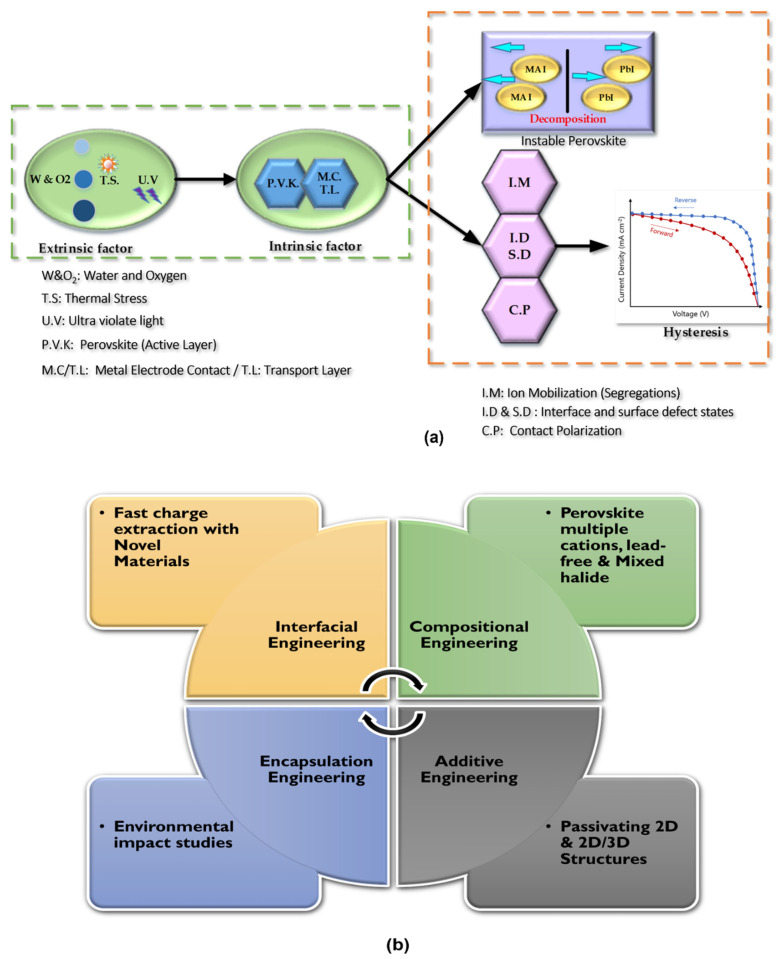
Schematic diagram of (**a**) the simple interplay between extrinsic and intrinsic perovskite performance degradation mechanisms with blue line arrows. The consequence of direct and indirect chain reactions is represented in red and green arrows, respectively. Hysteresis symbolic graph is depicted from Ref. [127]. (**b**) Summarizes the various perovskite engineering methods adopted in Table 4.

**Figure 7:**
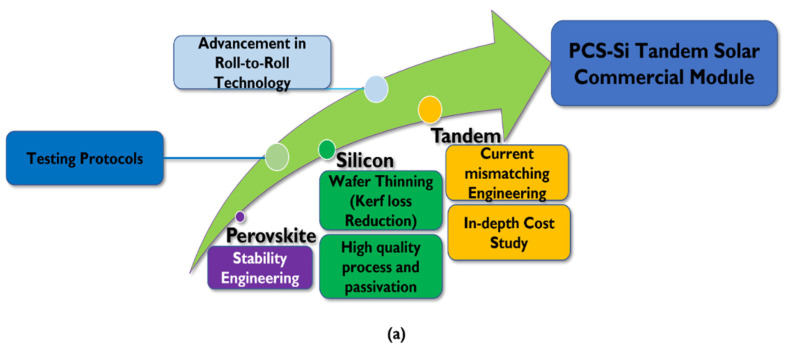

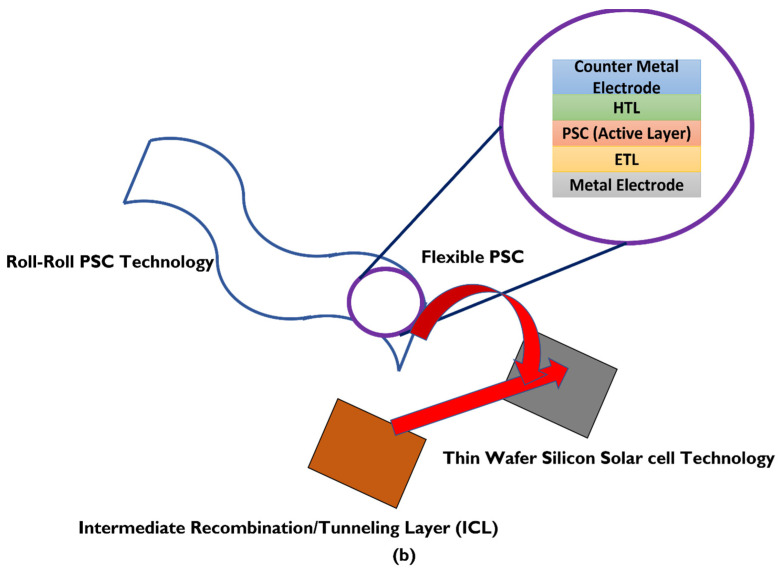
Schematic diagram of (**a**) A way forward towards PCS-Si tandem vast industrialization and the work required Table 2. T- PSC-Si based on roll-roll technology integration with existing silicon solar cell technology as part of the overall roadmap in (**b**).

Even though perovskite is a relatively cheap technology compared to silicon solar cell technology, its current reliability is not yet satisfied to uplift perovskite for large-scale industrialization [225,226]. Hence, various engineering such as fine compositional engineering, additive engineering, interface engineering, and inexpensive encapsulation engineering routes are required to optimize perovskite top-cell continuously, as shown under ‘stability engineering’ in Figure 4 and Figure 7.

Remarkably, for future perovskite module industrialization, the solution for processing the life cost cycle needs to have a shorter pay-back period than other solar cells [227]. In that sense, the scalable and promising alternative methods feasibly proven to commercialize perovskite are the sequential deposition process and the blading coating process. However, frequent observations showed anti-correlation between cell area and solar cell efficiency, which the scalable deposition methods need to consider [228,229].

Despite many efforts in establishing wide-scale deposition of contact technologies with industrial standards methods such as sputtering for perovskite transport layers [230,231,232,233,234,235], stagnating issues of stability and hysteresis remain major concerns [236,237,238]. Similarly, the co-evaporation deposition technique in Ref. [40] effectively preserves the bottom cell’s surface texturing. However, it does not fit in to be considered a high throughput industrial technique that requires a revolution in manufacturing technologies. Moreover, the lack of standardized testing protocols is cumbersome as perovskite testing protocols must be appropriately aligned with the established IEC-61215 [225,239]. Furthermore, Perovskite age-driven hysteresis testing protocols with actual in-situ conditions must be aligned with silicon photovoltaic-based protocols [240,241,242,243,244]. Ashraf U. et al. reported that the lack of perovskite solar cell standard test protocols resulted from an ambiguous way of stability record assessment [241,242]. 

Hence, inexpensive and reliable encapsulation may pave the way towards the elimination of the extrinsic instability factors. However, in the end, recent and economically effective encapsulation strategies elaborated in literature [243] have not yet fully considered the critical economic balance requirement for PSC-Si tandem’s sub-cell. This point is evident as silicon solar cell encapsulation has already been standardized. Therefore, any perovskite top cell tandem-based encapsulation methods must align with the existing bottom cell encapsulation techniques. Recently, a novel set of low lifetime testing protocols has been developed for perovskite [59]. Furthermore, current UV-filtered perovskite solar cell testing methods require a shift to a more standardized one, such as the International Electrotechnical Commission (IEC) testing protocols [44,225]. However, this is not the case to gain similar consumer confidence in silicon bottom cells, which render low lifetime testing conditions restricted to the research laboratory before realistic commercialization.

Although the roadmap for perovskite upscaling requires proper perovskite stability engineering (Figure 4b), the emerged microelectronic blade coating technology would have to be industrialized instead of the lab-based small-scale spin coating method, which lacks upscaling due to film non-uniformity [44,77,244]. Yaoguang et al. [225] briefed on the loss mechanisms, which pose a roadblock towards industrialization. However, in the same work, an explanation of the potential coating methods to transfer current mini and sub-module streams from laboratory-based efficiency to the in-situ modules was made. To further reduce LOCE of PSC-Si tandem solar cells, exploitation of perovskite roll-to-roll printing technologies could boost PSC-Silicon tandem research motives, as implemented by Toshiba for PSC cell technology [44,55]. With its laboratory-based successful mini-modules through blading technology, Toshiba’s perovskite solar cells have demonstrated cell efficiency of 10.5% at 5 cm^2^. Toshiba highlighted that one of the challenges in their upscaling methods from poly-crystalline perovskite film uniformity production and scribe process was the removal of the remnants from the top electrode during blade coating [81]. Even though the first goal was to introduce a novel and invariable meniscus organic methylammonium lead iodide material, the latter problem had been addressed through the optimized low-pressure blade coating in combination with undisclosed easily removable materials.

One step forward that would pave the road towards standardization has been claimed by Microquanta, a China-based company. Microquanta planned a 20 MW perovskite solar cell pilot line that has claimed the European Solar Test Installation Agency testing pass for 200 × 800 cm^2^ of PCE beyond 14% and 17% [245,246]. The current champion PSC-Si tandem cells are mostly tested in a controlled environment; thus, testing similar tandem cells at maximum power point (MPP) tracking is critical to certifying the feasibility of PSC-Si marketing.

The Anita Ho-Baillie research group at the University of Sydney (USYD) demonstrated a very optimistic process towards industrialization. The group adopted ITO or nanocrystalline-based silicon-free interfacial layer (tunneling) 2T monolithic technology to produce PSC-Si tandem over 21% at 16 cm^2^ without having to retool the current silicon single-junction solar cell technology [162,247,248,249]. The same group in earlier experiments demonstrated a 4 cm^2^ PSC-Si tandem cell by exploiting downshifting antireflective coating material, which acts as a UV filter and silicon light trapping layer [250]. Even though the overall work of the USYD group is promising, standardized protocols may need to be optimized in future days. Lastly, as the monolithic 2T tandem-based solar cells work towards vast industrialization, ICL engineering with highly conductive and optical index match deserves valuable engineering time such that the optoelectronic loss is negligible.

In the roll-roll context, assuming all aforementioned PSC-Si tandem cells stabilities and film uniformity (Table 2) are acceptable for IEC standards, we conceptually propose integrating perovskite roll-roll production line into existing silicon solar cells (Figure 7b). However, further investigation is required to align roll-roll perovskite technology with standard and futuristic thin wafer-silicon solar cells technology. Further, it may require a techno-economic evaluation, feasibility studies, and convincing equipment manufacturers to combine PSC and thin wafer silicon production lines.

With the rapid trend of perovskite solar cell’s PCE and the existing silicon solar cells, we expect to experience a decent shift in perovskite-silicon tandem future market share, provided that the significant challenges conveyed in this work and previous works are unraveled.

## 7. Conclusions

Perovskite-silicon tandem has a great chance to obtain decent market shares amongst anticipated PV tandems. However, the unprecedented development in the last seven years may require top cell tandem low-cost perovskite loss mechanism-related industrialization issues to be addressed.

The main challenges and proposed strategies of top cell perovskite to overcome loss mechanisms and their main mitigations methods were highlighted in this work. Perovskite instability, hysteresis, toxicity, and low-cost industrial film technologies are the main inter-related concerns for top cell industrialization. Furthermore, a cost-effective and atmospheric proof encapsulation requires more research to offset the extrinsic degradation effects. Despite the silicon bottom cell being a consolidated PV technology, the state-of-the-art silicon struggles to reach 27% efficiency. Therefore, critical passivation and light loss require more research. POLO-based silicon solar cell is another anticipated route for the bottom tandem solar cells due to the availability of the existing PERC production line.

A proposed roadmap based on the integration of existing perovskite roll-roll technology into silicon solar cells production line was presented in this work. Therefore, following the evolution of the proposed comprehensive perovskite stability engineering and testing protocols, the flexible and elastic substrate applications featuring roll-to-roll technologies may accommodate affordable and efficient PSC-Si tandem solar cells. As a result, wafer thinning, effective passivation, cost-effective light management techniques, and a shift in perovskite stability engineering can make a commercial PSC-Si tandem real. Similarly, advanced ICL layers require a more careful design and application, which needs special consideration on a large scale. Suppose the main perovskite-silicon tandem challenges towards broad marketing are solved. In that case, a revolution in the renewable energy PV market share may occur, especially for markets that opt-out to prevail during and after the COVID-19 pandemic period meeting the 2021 ITRPV projection.

## Figures and Tables

**Table 2:** Summary of the advantages and challenges based on PCS-Si tandem with various conditions, materials, and structure.

Tandem Solar Cell Type	Ref./Year	Advantage(s)	Limitation(s)
2T-PSC-Si	[35]/2020	- Champion cell, Highest PCE (29.15%), and FF (>80%) so far - Stable in overcoming PSC-hole carrier extraction efficiency with low ideality factor	- Current density mismatch (ΔJ) ~0.77 mA - Relative improvement of V_oc_ owed to non-radiative recombination at perovskite’s electron transport layer PSC-ETL
4T-PSC-Si	[36]/2020	- High PCE (28.3%) - Semitransparent Cr/Au/MgF_2_ front electrode allows IR light transmittance to the bottom cell (~66%)	- Slight Cr/Au light parasitic loss - Cr/Au/MgF_2_ Higher sheer resistivity which affected FF and J_sc_ of the top cell
2T-PSC-Si	[37]/2019	- PCE 26.0%, total J_sc_ 40mA. - Optimized layers thicknesses using Si homojunction bottom cell.	- Low fill factor (FF) - Higher current mismatch between sub-cells
2T-PSC-Si	[38]/2019	- PCE 25.2% @38.7 mA/cm^2^ via layers index matching techniques for flat surface silicon sub-cell	- PCE 25.2% is limited due to low FF
2T- Triple Junction-PSC/PSC/SHJ	[39]/2018	- High V_oc_ ~2.69 eV with optimized middle cell band gap with PSC sequential deposition technique	- PCE (14%) Limited due to low FF
2T PSC-Si	[40]/2018	- PCE 25.2% @19.5 mA @500 nm PSC @1.63 eV - Fully Textured cell. - Low lateral and conductive nc-Si: H - Device stability improved	- Requires more improvement in the front contact and successive layers. - Top cell voltage improvement towards wide-bandgap absorber. - Stability to be improved.
4T PSC-Si	[41]/2017	- An optimized 26.4% overall efficiency with PSC band gap 1.73eV with the use of Rubidium mixed-cations. - Improved cell stability and negligible hysteresis resulted from Rubidium	- Open circuit voltage is yet below the obtained bandgap with reduced FF
2T-PSC-Si	[42]/2017	- PCE 23.6% @1.63 eV V_oc_ - Reduced parasitic absorption loss - Enhanced stability through bilayer SnO_2_/ZTO as a diffusion barrier - Low-temperature deposition, i.e., ALD or pulsed-CVD over the rough surface of PSC - Silicon Nano Particles (SiNP) enhanced infrared EQE - Hysteresis free	- Due to PSC surface roughness, V_oc_ is low to reach the theoretical limit. - Front surface reflection still exists (to account for)
4T-PSC-Si	[32]/2016	- Improvement of perovskite aperture area to 1 cm^2^ from 0.25 cm^2^ total efficiency PCE of (23% and 25.2%) - n-i-p-based with PCE 20.5% monolithic at 1.43 cm is also developed in the same paper - Low-temperature PSC solvent deposition	- Low FF due to high series resistance in 1 cm^2^ cell - Parasitic absorption due to ITO and in Molybdenum oxide (MoO_x_) layers
2T PSC-Si	[43]/2015	- PCE 18.1% @1.78 V - Low Temperature deposition of ETL (SnO_2_) as replacement to the TiO_2_.	- Hall transport layer (HTL) Spiro-OMeTAD parasitic absorption with high overall reflectance and no surface texturing - ICL: ITO acts reflector

**Table 4:** The summaries of the significant stability sub-challenges with physical basis along with proposed healing/mitigation strategy (s).

No.	Challenge	Reasons	Remedies
1.	Moisture Instability	- Amine salt hydrophilicity [20]	- Mixed-cationic engineering with dual ion hybridization [82,95,96] - Low dimensionality perovskite and nanostructures integration [97,98]
2.	Oxygen instability	- Oxygen desorbing donor trapping sites [68] - May Water formation [99] - Superoxide formation [88,89]	- High binding energy layer/less oxygen reactive materials [82,89,90] - Cationic and passivation engineering [88,99]
3.	UV light instability	- Light-induced degradation of the constituent perovskite materials chemical instability, including charge transport layers [90,91]	- Proper interfacial engineering with barrier layers carrier transport [91,92]
			- Integration of 2D and 2D-3D composite perovskite [100]
			- Interface engineering such as in CsBr interface [101]
4.	Thermal instability	- De-phasing of the perovskite organic absorber due to thermal decomposition capacity of constituent’s materials (accelerated with light exposure) [102,103]	- Proper structural Alpha phase perovskite engineering (e.g., organic cationic engineering)
			- Surface engineering with organic cation termination and quantum dots Q.Ds [104]
			- 2D perovskite engineering [87,93]
		- Top layer thermal sensitivity [105]	- Novel electrode thermally stable materials [105]
5.	HTL and ETL interface instability	- Chemical reaction with environmental factors such as U.V and surrounding layers (e.g., TiO_2_) [106]	- Interface engineering with good blocking effect layers of diffused ions between Transport layer and perovskite modification [106]
		- Modification/irreversible deformation of HTL- Spiro-OMeTAD due to MA^+^ alteration, which may induce pinholes ready for iodide diffusion [107]	- Adoption of the improvement on the new concept materials such as stable transition metals oxides, carbon nanomaterials with modified interfacial layers (HTL Free) [108,109,110,111,112]
		- Li oxides doped-Spiro-OMeTAD salt hydrophilic and diffusive nature [83] - PEDOT: PSS high hygroscopes and acidity nature to react with Transparent Conductive Oxide (TCO) [82,83]	- Introduction of lithium-free dopants in the transport layers [83]
6.	Metal electrode layer-based instability	- Electrode interaction with the environment. Moreover, the Pinholes created with the interaction of metal contact with perovskite absorber-halide/transport layer may cause the formation of Aluminum/silver- halide compound (AlI_3_ and/or AgI) [84,85]	- Metal contact engineering with pinhole-free interfacial engineering (e.g., barrier design) [84] - 2D metal novel semitransparent doped materials (e.g., Graphene) [113,114,115,116]
7.	Processing-post process methods	- Low-temperature annealing process influence low fracture energy as a result of a small grain and rough films [117] - Water content during processing is responsible for increased hysteresis due to the increment of mobile ions [118] - Annealing temperature may lead to a difference in thermal coefficient resulted in inefficient interface connectivity [119] - Elimination of unreacted PbI_2_ [120]	- Understanding the interplay effect of optimum temperature and materials on perovskite thermomechanical properties and morphological aspects [117,121] - Solvent engineering using antisolvents helps create an intermediate phase that may remove humidity content after the annealing step [122] - Understanding of the influence of preprocessing and post-processing conditions and compositional engineering on the morphology and crystallinity of Perovskite [120]

## List of Acronyms

A	
Aluminum-Back Surface Field	Al-BSF
B	
Balance of the System	BoS
C	
Capital Expenditures	CapEx
Chemical Vapor Deposition	CVD
Coronavirus Disease 2019	COVID-19
D	
Double Anti Reflective Coating	DARC
E	
Electron Transport Layer	ETL
electron Volt	eV
EPFL	École polytechnique fédérale de Lausanne
Equation	EQ
F	
FA	Formamidinium
Fermi Level Pinning	FLP
Fill Factor	FF
Four Terminals	4T
H	
Hole Transport Layer	HTL
Indium-doped Tin Oxide	ITO
I	
IMEC	Interuniversity Microelectronics Centre
Indium-doped Zinc Oxide	IZO
Infrared	IR
Inter-digitated Back Contact	IBC
International Technology Roadmap for Photovoltaic	ITRPV
International Electrotechnical Commission	IEC
I/I^+^	Iodine/Iodide
J	
J-V	Current-Voltage
K	
kilo-Watt-hour	kWh
L	
Levelized Cost of generated Electricity	LCOE
Low-Pressure Chemical Vapor Deposition	LPCVD
M	
Milli-Ampere	mA
Molybdenum Oxide	MoO_x_
N	
nano-meter	nm
Nickel Oxide	NiOx
O	
Open circuit voltage	Voc
P	
Passivated Emitter And Rear Cell	PERC
Perovskite Solar Cell	PSC
Perovskite-Silicon Tandem Solar Cell	PSC-Si
Photovoltaic	PV
Plasma-Enhanced Chemical Vapor Deposition	PECVD
Polycrystalline Silicon On Oxide	POLO
Power Conversion Efficiencies	PCE
S	
Silicon Heterojunction Solar Cell	SHJ
Surface Recombination Velocity	SRV
T	
Tandem Solar Cell	TSC
Transition Metal Oxide	TMO
Transparent Conductive Oxide	TCO
Tunnel Oxide Passivated Contact	TOPCon
Two/Three Dimensional	2D/3D
Two Terminals	2T
U	
Ultraviolet	UV
University of Sydney	USYD

## References

[1] Covid-19 and the Clean Energy Challenges and Opportunities|Standard Chartered. https://www.sc.com/en/trade-beyond-borders/covid-19-clean-energy-challenges-and-opportunities/.

[2] Global Renewable Energy Market and the Affects of COVID-19 to 2021—The Solar Segment Dominates the Renewable Energy Sector. https://www.globenewswire.com/news-release/2020/05/05/2027415/0/en/Global-Renewable-Energy-Market-and-the-Affects-of-COVID-19-to-2021-The-Solar-Segment-Dominats-the-Renewable-Energy-Sector.html.

[3] Price-Efficiency Relationship for Photovoltaic Systems on a Global Basis. https://www.hindawi.com/journals/ijp/2015/256101/.

[4] Boriskina S.V., Chen G. (2014). Exceeding the solar cell Shockley-Queisser limit via thermal up-conversion of low-energy photons. Opt. Commun..

[5] Green M.A., Hishikawa Y., Dunlop E.D., Levi D.H., Hohl-Ebinger J., Ho-Baillie A.W.Y. (2018). Solar cell efficiency tables (version 52). Prog. Photovolt. Res. Appl..

[6] Yoshikawa K., Kawasaki H., Yoshida W., Irie T., Konishi K., Nakano K., Uto T., Adachi D., Kanematsu M., Uzu H. (2017). Silicon heterojunction solar cell with interdigitated back contacts for a photoconversion efficiency over 26%. Nat. Energy.

[7] Asim N., Sopian K., Ahmadi S., Saeedfar K., Alghoul M.A., Saadatian O., Zaidi S.H. (2012). A review on the role of materials science in solar cells. Renew. Sustain. Energy Rev..

[8] Detailed Balance Limit of the Efficiency of Tandem Solar Cells—IOP Science. https://iopscience.iop.org/article/10.1088/0022-3727/13/5/018/meta.

[9] Leijtens T., Bush K.A., Prasanna R., McGehee M.D. (2018). Opportunities and challenges for tandem solar cells using metal halide perovskite semiconductors. Nat. Energy.

[10] McMeekin D.P., Mahesh S., Noel N.K., Klug M.T., Lim J.C., Warby J.H., Ball J.M., Herz L.M., Johnston M.B., Snaith H.J. (2019). Solution-Processed All-Perovskite Multi-junction Solar Cells. Joule.

[11] Yamaguchi M., Lee K., Araki K. (2018). A review of recent progress in heterogeneous silicon tandem solar cells. J. Phys..

[12] Zulhafizhazuan W., Sepeai S., Siu Leong C., Sopian K., Zaidi S.H. (2017). Malaysian journal of analytical sciences performance analysis of simplified silicon solar cell on p-type crystalline silicon wafer. Malays. J. Anal. Sci..

[13] Hossain M.I., Qarony W., Ma S., Zeng L., Knipp D., Tsang Y.H. (2019). Perovskite/Silicon Tandem Solar Cells: From Detailed Balance Limit Calculations to Photon Management. Nano-Micro Lett..

[14] Yamaguchi M. Potential and present status of III-V/Si tandem solar cells. Proceedings of the 2014 IEEE 40th Photovoltaic Specialist Conference, PVSC 2014.

[15] Photovoltaic Roadmap (ITRPV): Eleventh Edition. https://itrpv.vdma.org/documents/105945/48393009/2020-04-28%2520PR%2520VDMA%2520PV%2520ITRPV%25202020%2520EN_1588057628144.pdf/87aba656-300c-c7a1-88c7-282f96a5dc6a.

[16] Lee T.D., Ebong A.U. (2017). A review of thin film solar cell technologies and challenges. Renew. Sustain. Energy Rev..

[17] Kour N., Mehra R. (2017). Recent Advances in Photovoltaic Technology based on Perovskite Solar Cell—A Review. Int. Res. J. Eng. Technol..

[18] Salhi B., Wudil Y.S., Hossain M.K., Al-Ahmed A., Al-Sulaiman F.A. (2018). Review of recent developments and persistent challenges in stability of perovskite solar cells. Renew. Sustain. Energy Rev..

[19] Krishnan U. (2019). Factors affecting the stability of perovskite solar cells: A comprehensive review. J. Photonics Energy.

[20] Wang R., Mujahid M., Duan Y., Wang Z.K., Xue J., Yang Y. (2019). A Review of Perovskites Solar Cell Stability. Adv. Funct. Mater..

[21] Yang Z., Babu B.H., Wu S., Liu T., Fang S., Xiong Z., Han L., Chen W. (2020). Review on Practical Interface Engineering of Perovskite Solar Cells: From Efficiency to Stability. Sol. RRL.

[22] Hermle M., Feldmann F., Bivour M., Goldschmidt J.C., Glunz S.W. (2020). Passivating contacts and tandem concepts: Approaches for the highest silicon-based solar cell efficiencies. Appl. Phys. Rev..

[23] Akhil S., Akash S., Pasha A., Kulkarni B., Jalalah M., Alsaiari M., Harraz F.A., Balakrishna R.G. (2021). Review on perovskite silicon tandem solar cells: Status and prospects 2T, 3T and 4T for real world conditions. Mater. Des..

[24] Wu T., Qin Z., Wang Y., Wu Y., Chen W., Zhang S., Cai M., Dai S., Zhang J., Liu J. (2021). The Main Progress of Perovskite Solar Cells in 2020–2021. Nano-Micro Lett..

[25] Liu N., Wang L., Xu F., Wu J., Song T., Chen Q. (2020). Recent Progress in Developing Monolithic Perovskite/Si Tandem Solar Cells. Front. Chem..

[26] Kim C.U., Jung E.D., Noh Y.W., Seo S.K., Choi Y., Park H., Song M.H., Choi K.J. (2021). Strategy for large-scale monolithic Perovskite/Silicon tandem solar cell: A review of recent progress. EcoMat.

[27] Sheng R., Hörantner M.T., Wang Z., Jiang Y., Zhang W., Agosti A., Huang S., Hao X., Ho-Baillie A., Green M. (2017). Monolithic Wide Band Gap Perovskite/Perovskite Tandem Solar Cells with Organic Recombination Layers. J. Phys. Chem. C.

[28] Cornet C., Da Silva M., Levallois C., Durand O. (2018). GaP/Si-based photovoltaic devices grown by molecular beam epitaxy. Molecular Beam Epitaxy.

[29] Meng L., Zhang Y., Wan X., Li C., Zhang X., Wang Y., Ke X., Xiao Z., Ding L., Xia R. (2018). Organic and solution-processed tandem solar cells with 17.3% efficiency. Science.

[30] Uzu H., Ichikawa M., Hino M., Nakano K., Meguro T., Hernández J.L., Kim H.S., Park N.G., Yamamoto K. (2015). High efficiency solar cells combining a perovskite and a silicon heterojunction solar cells via an optical splitting system. Appl. Phys. Lett..

[31] Sheng R., Ho-Baillie A.W.Y., Huang S., Keevers M., Hao X., Jiang L., Cheng Y.B., Green M.A. (2015). Four-Terminal Tandem Solar Cells Using CH_3_NH_3_PbBr_3_ by Spectrum Splitting. J. Phys. Chem. Lett..

[32] Werner J., Barraud L., Walter A., Bräuninger M., Sahli F., Sacchetto D., Tétreault N., Paviet-Salomon B., Moon S.J., Allebé C. (2016). Efficient Near-Infrared-Transparent Perovskite Solar Cells Enabling Direct Comparison of 4-Terminal and Monolithic Perovskite/Silicon Tandem Cells. ACS Energy Lett..

[33] Wali Q., Elumalai N.K., Iqbal Y., Uddin A., Jose R. (2018). Tandem perovskite solar cells. Renew. Sustain. Energy Rev..

[34] Eperon G.E., Leijtens T., Bush K.A., Prasanna R., Green T., Wang J.T.W., McMeekin D.P., Volonakis G., Milot R.L., May R. (2016). Perovskite-perovskite tandem photovoltaics with optimized band gaps. Science.

[35] Al-Ashouri A., Köhnen E., Li B., Magomedov A., Hempel H., Caprioglio P., Márquez J.A., Vilches A.B.M., Kasparavicius E., Smith J.A. (2020). Monolithic perovskite/silicon tandem solar cell with >29% efficiency by enhanced hole extraction. Science.

[36] Yang D., Zhang X., Hou Y., Wang K., Ye T., Yoon J., Wu C., Sanghadasa M., Liu S.F., Priya S. (2021). 28.3%-efficiency perovskite/silicon tandem solar cell by optimal transparent electrode for high efficient semitransparent top cell. Nano Energy.

[37] Köhnen E., Jošt M., Morales-Vilches A.B., Tockhorn P., Al-Ashouri A., Macco B., Kegelmann L., Korte L., Rech B., Schlatmann R. (2019). Highly efficient monolithic perovskite silicon tandem solar cells: Analyzing the influence of current mismatch on device performance. Sustain. Energy Fuels.

[38] Mazzarella L., Lin Y.H., Kirner S., Morales-Vilches A.B., Korte L., Albrecht S., Crossland E., Stannowski B., Case C., Snaith H.J. (2019). Infrared Light Management Using a Nanocrystalline Silicon Oxide Interlayer in Monolithic Perovskite/Silicon Heterojunction Tandem Solar Cells with Efficiency above 25%. Adv. Energy Mater..

[39] Werner J., Sahli F., Fu F., Diaz Leon J.J., Walter A., Kamino B.A., Niesen B., Nicolay S., Jeangros Q., Ballif C. (2018). Perovskite/Perovskite/Silicon Monolithic Triple-Junction Solar Cells with a Fully Textured Design. ACS Energy Lett..

[40] Sahli F., Werner J., Kamino B.A., Bräuninger M., Monnard R., Paviet-Salomon B., Barraud L., Ding L., Diaz Leon J.J., Sacchetto D. (2018). Fully textured monolithic perovskite/silicon tandem solar cells with 25.2% power conversion efficiency. Nat. Mater..

[41] Duong T., Wu Y.L., Shen H., Peng J., Fu X., Jacobs D., Wang E.C., Kho T.C., Fong K.C., Stocks M. (2017). Rubidium Multication Perovskite with Optimized Bandgap for Perovskite-Silicon Tandem with over 26% Efficiency. Adv. Energy Mater..

[42] Bush K.A., Palmstrom A.F., Yu Z.J., Boccard M., Cheacharoen R., Mailoa J.P., McMeekin D.P., Hoye R.L.Z., Bailie C.D., Leijtens T. (2017). 23.6%-efficient monolithic perovskite/silicon tandem solar cells with improved stability. Nat. Energy.

[43] Torabi N., Behjat A., Zhou Y., Docampo P., Stoddard R.J., Hillhouse H.W., Ameri T. (2019). Progress and challenges in perovskite photovoltaics from single- to multi-junction cells. Mater. Today Energy.

[44] Albrecht S., Saliba M., Correa Baena J.P., Lang F., Kegelmann L., Mews M., Steier L., Abate A., Rappich J., Korte L. (2016). Monolithic perovskite/silicon-heterojunction tandem solar cells processed at low temperature. Energy Environ. Sci..

[45] Lin R., Xiao K., Qin Z., Han Q., Zhang C., Wei M., Saidaminov M.I., Gao Y., Xu J., Xiao M. (2019). Monolithic all-perovskite tandem solar cells with 24.8% efficiency exploiting comproportionation to suppress Sn(ii) oxidation in precursor ink. Nat. Energy.

[46] Ramli N.F., Sepeai S., Rostan N.F.M., Ludin N.A., Ibrahim M.A., Teridi M.A.M., Zaidi S.H. (2017). Model development of monolithic tandem silicon-perovskite solar cell by SCAPS simulation. AIP Conference Proceedings.

[47] Jiang M., Deng N., Wang L., Xie H., Qiu Y. (2018). The structural, electronic, and optical properties of organic-inorganic mixed halide perovskites CH_3_NH_3_Pb(I_1-y_X_y_)_3_ (X = Cl, Br). Chin. Phys. B.

[48] Yu Z.J., Carpenter J.V., Holman Z.C. (2018). Techno-economic viability of silicon-based tandem photovoltaic modules in the United States. Nat. Energy.

[49] Chen B., Bai Y., Yu Z., Li T., Zheng X., Dong Q., Shen L., Boccard M., Gruverman A., Holman Z. (2016). Efficient Semitransparent Perovskite Solar Cells for 23.0%-Efficiency Perovskite/Silicon Four-Terminal Tandem Cells. Adv. Energy Mater..

[50] Perovskite on Silicon Tandem Solar Cell Technology|Oxford PV. https://www.oxfordpv.com/perovskite-silicon-tandem.

[51] Press Release—Imec Beats Silicon PV with 27.1 Percent Perovskite-Silicon Tandem. https://www.imec-int.com/en/articles/imec-beats-silicon-pv-with-27-1-percent-perovskite-silicon-tandem.

[52] World Record: Efficiency of Perovskite Silicon Tandem Solar Cell Jumps to 29.15 Percent. https://www.helmholtz-berlin.de/pubbin/news_seite?nid=21020;sprache=en;seitenid=72384.

[53] Lamanna E., Matteocci F., Calabrò E., Serenelli L., Salza E., Martini L., Menchini F., Izzi M., Agresti A., Pescetelli S. (2020). Mechanically Stacked, Two-Terminal Graphene-Based Perovskite/Silicon Tandem Solar Cell with Efficiency over 26%. Joule.

[54] Hou Y., Aydin E., De Bastiani M., Xiao C., Isikgor F.H., Xue D.-J., Chen B., Chen H., Bahrami B., Chowdhury A.H. (2020). Efficient tandem solar cells with solution-processed perovskite on textured crystalline silicon. Science.

[55] Li Z., Zhao Y., Wang X., Sun Y., Zhao Z., Li Y., Zhou H., Chen Q. (2018). Cost Analysis of Perovskite Tandem Photovoltaics. Joule.

[56] Bremner S.P., Levy M.Y., Honsberg C.B. (2008). Analysis of tandem solar cell efficiencies under AM1.5G spectrum using a rapid flux calculation method. Prog. Photovolt. Res. Appl..

[57] Hörantner M.T., Snaith H.J. (2017). Predicting and optimising the energy yield of perovskite-on-silicon tandem solar cells under real world conditions. Energy Environ. Sci..

[58] Correa-Baena J.P., Saliba M., Buonassisi T., Grätzel M., Abate A., Tress W., Hagfeldt A. (2017). Promises and challenges of perovskite solar cells. Science.

[59] Green M., Dunlop E., Hohl-Ebinger J., Yoshita M., Kopidakis N., Hao X. (2021). Solar cell efficiency tables (version 57). Prog. Photovolt. Res. Appl..

[60] Snaith H.J. (2018). Present status and future prospects of perovskite photovoltaics. Nat. Mater..

[61] Lal N.N., Dkhissi Y., Li W., Hou Q., Cheng Y.B., Bach U. (2017). Perovskite Tandem Solar Cells. Adv. Energy Mater..

[62] Banerjee D., Chattopadhyay K.K. (2018). Hybrid inorganic organic perovskites. Perovskite Photovoltaics: Basic to Advanced Concepts and Implementation.

[63] Yang Z., Yu Z., Wei H., Xiao X., Ni Z., Chen B., Deng Y., Habisreutinger S.N., Chen X., Wang K. (2019). Enhancing electron diffusion length in narrow-bandgap perovskites for efficient monolithic perovskite tandem solar cells. Nat. Commun..

[64] Zheng J., Mehrvarz H., Ma F.J., Lau C.F.J., Green M.A., Huang S., Ho-Baillie A.W.Y. (2018). 21.8% Efficient Monolithic Perovskite/Homo-Junction-Silicon Tandem Solar Cell on 16 cm^2^. ACS Energy Lett..

[65] Qarony W., Hossain M.I., Salleo A., Knipp D., Tsang Y.H. (2019). Rough versus planar interfaces: How to maximize the short circuit current of perovskite single and tandem solar cells. Mater. Today Energy.

[66] Mora-Seró I. (2018). How Do Perovskite Solar Cells Work?. Joule.

[67] Turren-Cruz S.H., Saliba M., Mayer M.T., Juárez-Santiesteban H., Mathew X., Nienhaus L., Tress W., Erodici M.P., Sher M.J., Bawendi M.G. (2018). Enhanced charge carrier mobility and lifetime suppress hysteresis and improve efficiency in planar perovskite solar cells. Energy Environ. Sci..

[68] Mesquita I., Andrade L., Mendes A. (2018). Perovskite solar cells: Materials, configurations and stability. Renew. Sustain. Energy Rev..

[69] Lee M.M., Teuscher J., Miyasaka T., Murakami T.N., Snaith H.J. (2012). Efficient hybrid solar cells based on meso-superstructured organometal halide perovskites. Science.

[70] Kim H.S., Lee C.R., Im J.H., Lee K.B., Moehl T., Marchioro A., Moon S.J., Humphry-Baker R., Yum J.H., Moser J.E. (2012). Lead iodide perovskite sensitized all-solid-state submicron thin film mesoscopic solar cell with efficiency exceeding 9%. Sci. Rep..

[71] Tidrow S.C. (2014). Mapping comparison of goldschmidt’s tolerance factor with perovskite structural conditions. Ferroelectrics.

[72] Jung E.H., Jeon N.J., Park E.Y., Moon C.S., Shin T.J., Yang T.Y., Noh J.H., Seo J. (2019). Efficient, stable and scalable perovskite solar cells using poly(3-hexylthiophene). Nature.

[73] Jiang Q., Chu Z., Wang P., Yang X., Liu H., Wang Y., Yin Z., Wu J., Zhang X., You J. (2017). Planar-Structure Perovskite Solar Cells with Efficiency beyond 21%. Adv. Mater..

[74] Wang Y., Zhang T., Kan M., Zhao Y. (2018). Bifunctional Stabilization of All-Inorganic α-CsPbI_3_ Perovskite for 17% Efficiency Photovoltaics. J. Am. Chem. Soc..

[75] Guenther K.M., Baumann A.L., Gimpel T., Kontermann S., Schade W. (2012). Tandem solar cell concept using Black Silicon for enhanced infrared absorption. Energy Procedia.

[76] Li Z., Klein T.R., Kim D.H., Yang M., Berry J.J., Zhu K. (2018). Scaling Up Perovskite Photovoltaics: Progress, Challenges, and Outlook of a Transformational Technology. Nat. Rev. Mater..

[77] Kim D.H., Whitaker J.B., Li Z., van Hest M.F.A.M., Zhu K. (2018). Outlook and Challenges of Perovskite Solar Cells toward Terawatt-Scale Photovoltaic Module Technology. Joule.

[78] Galagan Y., Coenen E.W.C., Verhees W.J.H., Andriessen R. (2016). Towards the scaling up of perovskite solar cells and modules. J. Mater. Chem. A.

[79] Schileo G., Grancini G. (2020). Halide perovskites: Current issues and new strategies to push material and device stability. J. Phys. Energy.

[80] Zhang S., Han G. (2020). Intrinsic and environmental stability issues of perovskite photovoltaics. Prog. Energy.

[81] Toshiba Corporate Research & Development Center Toshiba Achieves World’s Highest Conversion Efficiency in 5 cm × 5 cm Film-Based Perovskite Solar Cell Mini-Modules—Technology Will Reduce Cost and Increase Efficiency of Flexible Solar Cell Modules that Can Be Installed on Walls and Other Locations. https://www.toshiba.co.jp/rdc/rd/detail_e/e1709_02.html.

[82] Wang R.T., Tai E.G., Chen J.Y., Xu G., LaPierre R., Goktas N.I., Hu N. (2019). A KMnF 3 perovskite structure with improved stability, low bandgap and high transport properties. Ceram. Int..

[83] Schloemer T.H., Christians J.A., Luther J.M., Sellinger A. (2019). Doping strategies for small molecule organic hole-transport materials: Impacts on perovskite solar cell performance and stability. Chem. Sci..

[84] Kato Y., Ono L.K., Lee M.V., Wang S., Raga S.R., Qi Y. (2015). Silver Iodide Formation in Methyl Ammonium Lead Iodide Perovskite Solar Cells with Silver Top Electrodes. Adv. Mater. Interfaces.

[85] Guerrero A., You J., Aranda C., Kang Y.S., Garcia-Belmonte G., Zhou H., Bisquert J., Yang Y. (2016). Interfacial degradation of planar lead halide perovskite solar cells. ACS Nano.

[86] Min H., Kim G., Paik M.J., Lee S., Yang W.S., Jung M., Seok S.I. (2019). Stabilization of Precursor Solution and Perovskite Layer by Addition of Sulfur. Adv. Energy Mater..

[87] Lin Y., Bai Y., Fang Y., Chen Z., Yang S., Zheng X., Tang S., Liu Y., Zhao J., Huang J. (2018). Enhanced Thermal Stability in Perovskite Solar Cells by Assembling 2D/3D Stacking Structures. J. Phys. Chem. Lett..

[88] Aristidou N., Eames C., Sanchez-Molina I., Bu X., Kosco J., Saiful Islam M., Haque S.A. (2017). Fast oxygen diffusion and iodide defects mediate oxygen-induced degradation of perovskite solar cells. Nat. Commun..

[89] Aristidou N., Sanchez-Molina I., Chotchuangchutchaval T., Brown M., Martinez L., Rath T., Haque S.A. (2015). The Role of Oxygen in the Degradation of Methylammonium Lead Trihalide Perovskite Photoactive Layers. Angew. Chemie Int. Ed..

[90] Liu S.C., Li Z., Yang Y., Wang X., Chen Y.X., Xue D.J., Hu J.S. (2019). Investigation of Oxygen Passivation for High-Performance All-Inorganic Perovskite Solar Cells. J. Am. Chem. Soc..

[91] Niu G., Guo X., Wang L. (2015). Review of recent progress in chemical stability of perovskite solar cells. J. Mater. Chem. A.

[92] Leijtens T., Eperon G.E., Pathak S., Abate A., Lee M.M., Snaith H.J. (2013). Overcoming ultraviolet light instability of sensitized TiO_2_ with meso-superstructured organometal tri-halide perovskite solar cells. Nat. Commun..

[93] Arabpour Roghabadi F., Alidaei M., Mousavi S.M., Ashjari T., Tehrani A.S., Ahmadi V., Sadrameli S.M. (2019). Stability progress of perovskite solar cells dependent on the crystalline structure: From 3D ABX3 to 2D Ruddlesden-Popper perovskite absorbers. J. Mater. Chem. A.

[94] Qin X., Zhao Z., Wang Y., Wu J., Jiang Q., You J. (2017). Recent progress in stability of perovskite solar cells. J. Semicond..

[95] Boix P.P., Agarwala S., Koh T.M., Mathews N., Mhaisalkar S.G. (2015). Perovskite solar cells: Beyond methylammonium lead iodide. J. Phys. Chem. Lett..

[96] Maniarasu S., Korukonda T.B., Manjunath V., Ramasamy E., Ramesh M., Veerappan G. (2018). Recent advancement in metal cathode and hole-conductor-free perovskite solar cells for low-cost and high stability: A route towards commercialization. Renew. Sustain. Energy Rev..

[97] Petrović M., Rogdakis K., Kymakis E. (2019). Beneficial impact of materials with reduced dimensionality on the stability of perovskite-based photovoltaics. J. Phys. Energy.

[98] Poli I., Liang X., Baker R., Eslava S., Cameron P.J. (2018). Enhancing the hydrophobicity of perovskite solar cells using C18 capped CH_3_NH_3_PbI_3_ nanocrystals. J. Mater. Chem. C.

[99] Senocrate A., Acartürk T., Kim G.Y., Merkle R., Starke U., Grätzel M., Maier J. (2018). Interaction of oxygen with halide perovskites. J. Mater. Chem. A.

[100] Zhao L., Tian H., Silver S.H., Kahn A., Ren T.L., Rand B.P. (2018). Ultrasensitive Heterojunctions of Graphene and 2D Perovskites Reveal Spontaneous Iodide Loss. Joule.

[101] Li W., Zhang W., van Reenen S., Sutton R.J., Fan J., Haghighirad A.A., Johnston M.B., Wang L., Snaith H.J. (2016). Enhanced UV-light stability of planar heterojunction perovskite solar cells with caesium bromide interface modification. Energy Environ. Sci..

[102] Conings B., Drijkoningen J., Gauquelin N., Babayigit A., D’Haen J., D’Olieslaeger L., Ethirajan A., Verbeeck J., Manca J., Mosconi E. (2015). Intrinsic Thermal Instability of Methylammonium Lead Trihalide Perovskite. Adv. Energy Mater..

[103] Dualeh A., Gao P., Seok S.I., Nazeeruddin M.K., Grätzel M. (2014). Thermal behavior of methylammonium lead-trihalide perovskite photovoltaic light harvesters. Chem. Mater..

[104] Wang Z., Baranwal A.K., Kamarudin M.A., Kamata Y., Ng C.H., Pandey M., Ma T., Hayase S. (2019). Structured crystallization for efficient all-inorganic perovskite solar cells with high phase stability. J. Mater. Chem. A.

[105] Liu Z., Li J., Sun Z.H., Tai G., Lau S.P., Yan F. (2012). The application of highly doped single-layer graphene as the top electrodes of semitransparent organic solar cells. ACS Nano.

[106] Rong Y., Liu L., Mei A., Li X., Han H. (2015). Beyond efficiency: The challenge of stability in mesoscopic perovskite solar cells. Adv. Energy Mater..

[107] Jena A.K., Numata Y., Ikegami M., Miyasaka T. (2018). Role of spiro-OMeTAD in performance deterioration of perovskite solar cells at high temperature and reuse of the perovskite films to avoid Pb-waste. J. Mater. Chem. A.

[108] Wang K., Olthof S., Subhani W.S., Jiang X., Cao Y., Duan L., Wang H., Du M., Liu S. (2020). (Frank) Novel inorganic electron transport layers for planar perovskite solar cells: Progress and prospective. Nano Energy.

[109] Mohamad Noh M.F., Teh C.H., Daik R., Lim E.L., Yap C.C., Ibrahim M.A., Ahmad Ludin N., Mohd Yusoff A.R.B., Jang J., Mat Teridi M.A. (2018). The architecture of the electron transport layer for a perovskite solar cell. J. Mater. Chem. C.

[110] Oo T.T., Debnath S. (2017). Application of carbon nanotubes in perovskite solar cells: A review. AIP Conference Proceedings.

[111] Thakur U.K., Kisslinger R., Shankar K. (2017). One-dimensional electron transport layers for perovskite solar cells. Nanomaterials.

[112] Kung P., Li M., Lin P., Chiang Y., Chan C., Guo T., Chen P. (2018). A Review of Inorganic Hole Transport Materials for Perovskite Solar Cells. Adv. Mater. Interfaces.

[113] Zhang H., Liu H., Lu W., Zhang W., Hao Y., Wang P., Yu W. (2019). Analysis and suppression of metal-contact-induced degradation in inverted triple cation perovskite solar cells. Mater. Lett..

[114] Behrouznejad F., Shahbazi S., Taghavinia N., Wu H.P., Wei-Guang Diau E. (2016). A study on utilizing different metals as the back contact of CH_3_NH_3_PbI_3_ perovskite solar cells. J. Mater. Chem. A.

[115] Boyd C.C., Cheacharoen R., Bush K.A., Prasanna R., Leijtens T., McGehee M.D. (2018). Barrier Design to Prevent Metal-Induced Degradation and Improve Thermal Stability in Perovskite Solar Cells. ACS Energy Lett..

[116] Lee K.T., Park D.H., Baac H.W., Han S. (2018). Graphene- and carbon-nanotube-based transparent electrodes for semitransparent solar cells. Materials.

[117] Rolston N., Watson B.L., Bailie C.D., McGehee M.D., Bastos J.P., Gehlhaar R., Kim J.E., Vak D., Mallajosyula A.T., Gupta G. (2016). Mechanical integrity of solution-processed perovskite solar cells. Extrem. Mech. Lett..

[118] Clegg C., Hill I.G. (2016). Systematic study on the impact of water on the performance and stability of perovskite solar cells. RSC Adv..

[119] Cojocaru L., Uchida S., Sanehira Y., Gonzalez-Pedro V., Bisquert J., Nakazaki J., Kubo T., Segawa H. (2015). Temperature Effects on the Photovoltaic Performance of Planar Structure Perovskite Solar Cells Advance Publication Cover Page Temperature Effects on the Photovoltaic Performance of Planar Structure Perovskite Solar Cells. Chem. Lett..

[120] Chen L.C., Lee K.L., Wu W.T., Hsu C.F., Tseng Z.L., Sun X.H., Kao Y.T. (2018). Effect of Different CH_3_NH_3_PbI_3_ Morphologies on Photovoltaic Properties of Perovskite Solar Cells. Nanoscale Res. Lett..

[121] The Effect of Grain Orientation on the Morphological Stability of the Organic–Inorganic Perovskite Films under Elevated Temperature—IOP Science. https://iopscience.iop.org/article/10.1088/1674-4926/38/1/014002/meta.

[122] Troughton J., Hooper K., Watson T.M. (2017). Humidity resistant fabrication of CH_3_NH_3_PbI_3_ perovskite solar cells and modules. Nano Energy.

[123] Fu Q., Tang X., Huang B., Hu T., Tan L., Chen L., Chen Y. (2018). Recent Progress on the Long-Term Stability of Perovskite Solar Cells. Adv. Sci..

[124] Fedorovskiy A.E., Drigo N.A., Nazeeruddin M.K. (2020). The Role of Goldschmidt’s Tolerance Factor in the Formation of A_2_BX_6_ Double Halide Perovskites and its Optimal Range. Small Methods.

[125] Sato T., Takagi S., Deledda S., Hauback B.C., Orimo S.I. (2016). Extending the applicability of the Goldschmidt tolerance factor to arbitrary ionic compounds. Sci. Rep..

[126] Bartel C.J., Sutton C., Goldsmith B.R., Ouyang R., Musgrave C.B., Ghiringhelli L.M., Scheffler M. (2019). New tolerance factor to predict the stability of perovskite oxides and halides. Sci. Adv..

[127] Weber S.A.L., Hermes I.M., Turren-Cruz S.H., Gort C., Bergmann V.W., Gilson L., Hagfeldt A., Graetzel M., Tress W., Berger R. (2018). How the formation of interfacial charge causes hysteresis in perovskite solar cells. Energy Environ. Sci..

[128] Morris K.A. (2011). What is hysteresis?. Appl. Mech. Rev..

[129] Berg S., Särhammar E., Nyberg T. (2014). Upgrading the “berg-model” for reactive sputtering processes. Thin Solid Films.

[130] Liu P., Wang W., Liu S., Yang H., Shao Z. (2019). Fundamental Understanding of Photocurrent Hysteresis in Perovskite Solar Cells. Adv. Energy Mater..

[131] Shi Z., Jayatissa A.H. (2018). Perovskites-based solar cells: A review of recent progress, materials and processing methods. Materials.

[132] Caram J., García-Batlle M., Almora O., Arce R.D., Guerrero A., Garcia-Belmonte G. (2020). Direct observation of surface polarization at hybrid perovskite/Au interfaces by dark transient experiments. Appl. Phys. Lett..

[133] Chen B., Rudd P.N., Yang S., Yuan Y., Huang J. (2019). Imperfections and their passivation in halide perovskite solar cells. Chem. Soc. Rev..

[134] Zhao P., Kim B.J., Jung H.S. (2018). Passivation in perovskite solar cells: A review. Mater. Today Energy.

[135] Chen W., Sun K., Ma C., Leng C., Fu J., Hu L., Li M., Wang M., Zang Z., Tang X. (2018). Eliminating J-V hysteresis in perovskite solar cells via defect controlling. Org. Electron..

[136] Lee J.W., Kim S.G., Yang J.M., Yang Y., Park N.G. (2019). Verification and mitigation of ion migration in perovskite solar cells. APL Mater..

[137] Futscher M.H., Lee J.M., McGovern L., Muscarella L.A., Wang T., Haider M.I., Fakharuddin A., Schmidt-Mende L., Ehrler B. (2019). Quantification of ion migration in CH_3_NH_3_PbI_3_ perovskite solar cells by transient capacitance measurements. Mater. Horizons.

[138] Gualdrón-Reyes A.F., Yoon S.J., Mora-Seró I. (2018). Recent insights for achieving mixed halide perovskites without halide segregation. Curr. Opin. Electrochem..

[139] Yoon S.J., Kuno M., Kamat P.V. (2017). Shift Happens. How Halide Ion Defects Influence Photoinduced Segregation in Mixed Halide Perovskites. ACS Energy Lett..

[140] Zhang H., Fu X., Tang Y., Wang H., Zhang C., Yu W.W., Wang X., Zhang Y., Xiao M. (2019). Phase segregation due to ion migration in all-inorganic mixed-halide perovskite nanocrystals. Nat. Commun..

[141] Brennan M.C., Draguta S., Kamat P.V., Kuno M. (2018). Light-Induced Anion Phase Segregation in Mixed Halide Perovskites. ACS Energy Lett..

[142] Elmelund T., Seger B., Kuno M., Kamat P.V. (2020). How Interplay between Photo and Thermal Activation Dictates Halide Ion Segregation in Mixed Halide Perovskites. ACS Energy Lett..

[143] Mahapatra A., Prochowicz D., Tavakoli M.M., Trivedi S., Kumar P., Yadav P. (2019). A review of aspects of additive engineering in perovskite solar cells. J. Mater. Chem. A.

[144] Influence of Surface States on Current-Voltage Characteristics of M-I-pSi Solar Cells—IOPscience. https://iopscience.iop.org/article/10.1088/0022-3727/25/1/014.

[145] Spicer W.E., Newman N., Spindt C.J., Liliental-Weber Z., Weber E.R. (1990). “Pinning” and Fermi level movement at GaAs surfaces and interfaces. J. Vac. Sci. Technol. A Vacuum Surfaces Film..

[146] Tengstedt C., Osikowicz W., Salaneck W.R., Parker I.D., Hsu C.H., Fahlman M. (2006). Fermi-level pinning at conjugated polymer interfaces. Appl. Phys. Lett..

[147] Zu F., Amsalem P., Ralaiarisoa M., Schultz T., Schlesinger R., Koch N. (2017). Surface State Density Determines the Energy Level Alignment at Hybrid Perovskite/Electron Acceptors Interfaces. ACS Appl. Mater. Interfaces.

[148] Gallet T., Grabowski D., Kirchartz T., Redinger A. (2019). Fermi-level pinning in methylammonium lead iodide perovskites. Nanoscale.

[149] Caprioglio P., Stolterfoht M., Wolff C.M., Unold T., Rech B., Albrecht S., Neher D. (2019). On the Relation between the Open-Circuit Voltage and Quasi-Fermi Level Splitting in Efficient Perovskite Solar Cells. Adv. Energy Mater..

[150] Jiang C., Zhang P. (2018). Crystalline orientation dependent photoresponse and heterogeneous behaviors of grain boundaries in perovskite solar cells. J. Appl. Phys..

[151] Caprioglio P., Zu F., Wolff C., Marquez-Prieto J., Berlin H. (2018). High Open Circuit Voltages in Pin-Type Perovskite Solar Cells through Strontium Addition. Sustain. Energy Fuels.

[152] Abdy H., Aletayeb A., Kolahdouz M., Soleimani E.A. (2019). Investigation of metal-nickel oxide contacts used for perovskite solar cell. AIP Adv..

[153] Soucase B.M., Guaita Pradas I., Adhikari K.R. (2016). Numerical Simulations on Perovskite Photovoltaic Devices. Perovskite Mater. Synth. Charact. Prop. Appl..

[154] Monteiro Lunardi M., Wing Yi Ho-Baillie A., Alvarez-Gaitan J.P., Moore S., Corkish R. (2017). A life cycle assessment of perovskite/silicon tandem solar cells. Prog. Photovolt. Res. Appl..

[155] Chen B., Fei C., Chen S., Gu H., Xiao X., Huang J. (2021). Recycling lead and transparent conductors from perovskite solar modules. Nat. Commun..

[156] Glunz S.W., Preu R., Biro D. (2012). Crystalline Silicon solar cells. State-of-the-art and future developments. Comprehensive Renewable Energy.

[157] Sergeev A., Sablon K. (2017). Shockley-Queisser Model: Analytical Solution, Thermodynamics, and Kinetics. arXiv.

[158] Swanson R.M. Approaching the 29% limit efficiency of silicon solar cells. Proceedings of the Conference Record of the IEEE Photovoltaic Specialists Conference.

[159] Rühle S. (2016). Tabulated values of the Shockley-Queisser limit for single junction solar cells. Sol. Energy.

[160] Almosni S., Delamarre A., Jehl Z., Suchet D., Cojocaru L., Giteau M., Behaghel B., Julian A., Ibrahim C., Tatry L. (2018). Material challenges for solar cells in the twenty-first century: Directions in emerging technologies. Sci. Technol. Adv. Mater..

[161] Luceño-Sánchez J.A., Díez-Pascual A.M., Capilla R.P. (2019). Materials for photovoltaics: State of art and recent developments. Int. J. Mol. Sci..

[162] Kumar A., Bieri M., Reindl T., Aberle A.G. (2017). Economic Viability Analysis of Silicon Solar Cell Manufacturing: Al-BSF versus PERC. Energy Procedia.

[163] Fu R., Feldman D.J., Margolis R.M. (2021). Solar Photovoltaic System Cost Benchmark: Q1 2018.

[164] Deb S.K. (1996). Thin-film solar cells: An overview. Renew. Energy.

[165] Wilson G.M., Al-Jassim M., Metzger W.K., Glunz S.W., Verlinden P., Xiong G., Mansfield L.M., Stanbery B.J., Zhu K., Yan Y. (2020). The 2020 Photovoltaic Technologies Roadmap. J. Phys. D.

[166] Zahidi S. From Carbon to Silicon: A Paradigm Shift in Energy Generation. http://ukmpress.ukm.my/index.php?route=product/product&product_id=470.

[167] Goetzberger A., Luther J., Willeke G. (2002). Solar cells: Past, present, future. Sol. Energy Mater. Sol. Cells.

[168] Fonash S.J. (2010). Solar Cell Device Physics.

[169] Soley S.S., Dwivedi A.D.D. (2018). Advances in high efficiency crystalline silicon homo junction solar cell technology. AIP Conference Proceedings.

[170] Tockhorn P., Wagner P., Kegelmann L., Stang J.C., Mews M., Albrecht S., Korte L. (2020). Three-Terminal Perovskite/Silicon Tandem Solar Cells with Top and Interdigitated Rear Contacts. ACS Appl. Energy Mater..

[171] Liu Z., Sofia S.E., Laine H.S., Woodhouse M., Wieghold S., Peters I.M., Buonassisi T. (2019). Revisiting Thin Silicon for Photovoltaics: A Technoeconomic Perspective. Energy Environ. Sci..

[172] Sai H., Oku T., Sato Y., Tanabe M., Matsui T., Matsubara K. (2019). Potential of very thin and high-efficiency silicon heterojunction solar cells. Prog. Photovolt. Res. Appl..

[173] Louwen A., van Sark W., Schropp R., Faaij A. (2016). A cost roadmap for silicon heterojunction solar cells. Sol. Energy Mater. Sol. Cells.

[174] Haase F., Hollemann C., Schäfer S., Merkle A., Rienäcker M., Krügener J., Brendel R., Peibst R. (2018). Laser contact openings for local poly-Si-metal contacts enabling 26.1%-efficient POLO-IBC solar cells. Sol. Energy Mater. Sol. Cells.

[175] Hollemann C., Haase F., Rienäcker M., Barnscheidt V., Krügener J., Folchert N., Brendel R., Richter S., Großer S., Sauter E. (2020). Separating the two polarities of the POLO contacts of an 26.1%-efficient IBC solar cell. Sci. Rep..

[176] Yoshikawa K., Yoshida W., Irie T., Kawasaki H., Konishi K., Ishibashi H., Asatani T., Adachi D., Kanematsu M., Uzu H. (2017). Exceeding conversion efficiency of 26% by heterojunction interdigitated back contact solar cell with thin film Si technology. Sol. Energy Mater. Sol. Cells.

[177] Richter A., Benick J., Feldmann F., Fell A., Hermle M., Glunz S.W. (2017). n-Type Si solar cells with passivating electron contact: Identifying sources for efficiency limitations by wafer thickness and resistivity variation. Sol. Energy Mater. Sol. Cells.

[178] Pascual Sánchez D., Puigdollers Codirector J., López J.M. (2015). Crystalline Silicon Heterojunction Solar Cells.

[179] Melskens J., van de Loo B.W.H., Macco B., Black L.E., Smit S., Kessels W.M.M. (2018). Passivating Contacts for Crystalline Silicon Solar Cells: From Concepts and Materials to Prospects. IEEE J. Photovolt..

[180] Bivour M. (2015). Silicon Heterojunction Solar Cells: Analysis and Basic Understanding.

[181] Yu C., Xu S., Yao J., Han S. (2018). Recent advances in and new perspectives on crystalline silicon solar cells with carrier-selective passivation contacts. Crystals.

[182] Cho J., Melskens J., Debucquoy M., Recamán Payo M., Jambaldinni S., Bearda T., Gordon I., Szlufcik J., Kessels W.M.M., Poortmans J. (2018). Passivating electron-selective contacts for silicon solar cells based on an a-Si:H/TiO *_x_* stack and a low work function metal. Prog. Photovolt. Res. Appl..

[183] Elsmani M.I., Cho J., Recaman Payo M., Gordon I., Tresso E., Fatima N., Adib Ibrahim M., Sopian K., Poortmans J. Analysis of the Interface of RF-Sputtered MoOx based Hole-Selective Contacts for Silicon Heterojunction Solar Cells. 37th European PV Solar Energy Conference and Exhibition (EU PVSEC).

[184] Wyrsch N. Advanced Light-Trapping in Thin-Film Silicon Solar Cells. Proceedings of the Optics for Solar Energy 2012.

[185] Andreani L.C., Bozzola A., Kowalczewski P., Liscidini M., Redorici L. (2019). Silicon solar cells: Toward the efficiency limits. Adv. Phys. X.

[186] Mat Desa M.K., Sapeai S., Azhari A.W., Sopian K., Sulaiman M.Y., Amin N., Zaidi S.H. (2016). Silicon back contact solar cell configuration: A pathway towards higher efficiency. Renew. Sustain. Energy Rev..

[187] Müller M., Fischer G., Bitnar B., Steckemetz S., Schiepe R., Mühlbauer M., Köhler R., Richter P., Kusterer C., Oehlke A. (2017). Loss analysis of 22% efficient industrial PERC solar cells. Energy Procedia.

[188] Zeman M., Zhang D. (2012). Heterojunction Silicon Based Solar Cells. Physics and Technology of Amorphous-Crystalline Heterostructure Silicon Solar Cells.

[189] Schmidt J., Peibst R., Brendel R. (2018). Surface passivation of crystalline silicon solar cells: Present and future. Sol. Energy Mater. Sol. Cells.

[190] Allen T.G., Bullock J., Yang X., Javey A., De Wolf S. (2019). Passivating contacts for crystalline silicon solar cells. Nat. Energy.

[191] Hossain M.J., Gregory G., Patel H., Guo S., Schneller E.J., Gabor A.M., Yang Z., Blum A.L., Davis K.O. Detailed Performance Loss Analysis of Silicon Solar Cells using High-Throughput Metrology Methods. Proceedings of the 2018 IEEE 7th World Conference on Photovoltaic Energy Conversion, WCPEC 2018—A Joint Conference of 45th IEEE PVSC, 28th PVSEC and 34th EU PVSEC.

[192] Gerling L.G., Mahato S., Voz C., Alcubilla R., Puigdollers J. (2015). Characterization of transition metal oxide/silicon heterojunctions for solar cell applications. Appl. Sci..

[193] Li F., Zhou Y., Liu M., Dong G., Liu F., Wang W., Yu D. (2019). Molybdenum oxide hole selective transport layer by hot wire oxidation-sublimation deposition for silicon heterojunction solar cells. arXiv.

[194] Paviet-Salomon B., Tomasi A., Descoeudres A., Barraud L., Nicolay S., Despeisse M., Wolf S., De Ballif C. (2015). Back-Contacted Silicon Heterojunction Solar Cells: Optical-Loss Analysis and Mitigation. IEEE J. Photovolt..

[195] Park H., Lee Y.J., Park J., Kim Y., Yi J., Lee Y., Kim S., Park C.K., Lim K.J. (2018). Front and Back TCO Research Review of a-Si/c-Si Heterojunction with Intrinsic Thin Layer (HIT) Solar Cell. Trans. Electr. Electron. Mater..

[196] Cruz A., Berlin H., Neubert S., Berlin H., Berlin H., Erfurt D., Berlin H. Optoelectronic Performance of TCO on Silicon Heterojunction Rear-Emitter Solar Cells. Proceedings of the 35th European Photovoltaic Solar Energy Conference and Exhibition.

[197] Das S., Pandey D., Thomas J., Roy T. (2019). The Role of Graphene and Other 2D Materials in Solar Photovoltaics. Adv. Mater..

[198] Khanna A., Mueller T., Stangl R.A., Hoex B., Basu P.K., Aberle A.G. (2013). A fill factor loss analysis method for silicon wafer solar cells. IEEE J. Photovolt..

[199] Leilaeioun M.A. (2018). Fill Factor Loss Mechanisms: Analysis and Basic Understanding in Silicon Hetero-junction Solar Cells. Ph.D. Thesis.

[200] Gogolin R., Turcu M., Ferre R., Clemens J., Harder N.P., Brendel R., Schmidt J. (2014). Analysis of series resistance losses in a-Si:H/c-Si heterojunction solar cells. IEEE J. Photovolt..

[201] Li X., Yang L., Zhang W., Wang Q. (2019). Study the J SC loss of full area SHJ solar cells caused by edge recombination. J. Renew. Sustain. Energy.

[202] Elumalai N.K., Uddin A. (2016). Open circuit voltage of organic solar cells: An in-depth review. Energy Environ. Sci..

[203] DeWolf S., Descoeudres A., Holman Z.C., Ballif C. (2012). High-efficiency silicon heterojunction solar cells: A review. Green.

[204] Dullweber T., Kranz C., Peibst R., Baumann U., Hannebauer H., Fülle A., Steckemetz S., Weber T., Kutzer M., Müller M. (2016). PERC+: Industrial PERC solar cells with rear Al grid enabling bifaciality and reduced Al paste consumption. Prog. Photovolt. Res. Appl..

[205] PERC Market to Reach 158 GW by 2022—Pv Magazine International. https://www.pv-magazine.com/2018/09/07/perc-market-to-reach-158-gw-by-2022/.

[206] What Are Heterojunction Technology (HJT) Solar Panels?. https://www.solarpowerworldonline.com/2019/11/what-are-heterojunction-technology-hjt-solar-panels/.

[207] ITRPV ITRPV Road Map. https://itrpv.vdma.org/documents/27094228/29066965/ITRPV02020.pdf/ba3da187-3186-83de-784e-6e3b10d96f3f.

[208] Grancini G., Roldán-Carmona C., Zimmermann I., Mosconi E., Lee X., Martineau D., Narbey S., Oswald F., De Angelis F., Graetzel M. (2017). One-Year stable perovskite solar cells by 2D/3D interface engineering. Nat. Commun..

[209] Sun W., Choy K.L., Wang M. (2019). The role of thickness control and interface modification in assembling efficient planar perovskite solar cells. Molecules.

[210] Todorov T., Gershon T., Gunawan O., Sturdevant C., Guha S. (2014). Perovskite-kesterite monolithic tandem solar cells with high open-circuit voltage. Appl. Phys. Lett..

[211] Liu D., Gangishetty M.K., Kelly T.L. (2014). Effect of CH_3_NH_3_PbI_3_ thickness on device efficiency in planar heterojunction perovskite solar cells. J. Mater. Chem. A.

[212] Xi J., Wu Z., Dong H., Xia B., Yuan F., Jiao B., Xiao L., Gong Q., Hou X. (2015). Controlled thickness and morphology for highly efficient inverted planar heterojunction perovskite solar cells. Nanoscale.

[213] Swartwout R., Hoerantner M.T., Bulović V. (2019). Scalable Deposition Methods for Large Area Production of Perovskite Thin Films. Energy Environ. Mater..

[214] Das S., Gu G., Joshi P.C., Yang B., Aytug T., Rouleau C.M., Geohegan D.B., Xiao K. (2016). Low thermal budget, photonic-cured compact TiO_2_ layers for high-efficiency perovskite solar cells. J. Mater. Chem. A.

[215] Woodhouse M., Smith B., Ramdas A., Margolis R. (2019). Crystalline Silicon Photovoltaic Module Manufacturing Costs and Sustainable Pricing: 1H 2018 Benchmark and Cost Reduction Road Map.

[216] Sahli F., Kamino B.A., Werner J., Bräuninger M., Paviet-Salomon B., Barraud L., Monnard R., Seif J.P., Tomasi A., Jeangros Q. (2018). Improved Optics in Monolithic Perovskite/Silicon Tandem Solar Cells with a Nanocrystalline Silicon Recombination Junction. Adv. Energy Mater..

[217] Mazzarella L., Werth M., Jäger K., Jošt M., Korte L., Albrecht S., Schlatmann R., Stannowski B. (2018). Infrared photocurrent management in monolithic perovskite/silicon heterojunction tandem solar cells by using a nanocrystalline silicon oxide interlayer. Opt. Express.

[218] Heo J.H., Im S.H. (2016). CH_3_NH_3_PbBr_3_–CH_3_NH_3_PbI_3_Perovskite–Perovskite Tandem Solar Cells with Exceeding 2.2 V Open Circuit Voltage. Adv. Mater..

[219] Altazin S., Stepanova L., Werner J., Niesen B., Ballif C., Ruhstaller B. (2018). Design of perovskite/crystalline-silicon monolithic tandem solar cells. Opt. Express.

[220] Abate A., Correa-Baena J.-P., Saliba M., Su’ait M.S., Bella F. (2018). Perovskite Solar Cells: From the Laboratory to the Assembly Line. Chem. A Eur. J..

[221] Perovskite/Silicon Tandems for a High Performance|TNO. https://www.tno.nl/en/focus-areas/energy-transition/roadmaps/towards-ubiquitous-solar-energy/global-leader-with-innovative-solar-panels/perovskite-silicon-tandems-for-a-high-performance/.

[222] Bonilla R.S., Hoex B., Wilshaw P.R. (2020). Special issue: Surface and interface passivation in crystalline silicon solar cells. Sol. Energy Mater. Sol. Cells.

[223] Ur Rehman A., Iqbal M.Z., Bhopal M.F., Khan M.F., Hussain F., Iqbal J., Khan M., Lee S.H. (2018). Development and prospects of surface passivation schemes for high-efficiency c-Si solar cells. Sol. Energy.

[224] nanoGe—NIPHO20—Toward Industrialization of Monolithic Perovskite/Silicon-Heterojunction Tandem Solar Cells: Screen-Printing Metallization Development. https://www.nanoge.org/proceedings/NIPHO20/5dc438c3a7d38c1ffa0167ab.

[225] Rong Y., Hu Y., Mei A., Tan H., Saidaminov M.I., Seok S.I., McGehee M.D., Sargent E.H., Han H. (2018). Challenges for commercializing perovskite solar cells. Science.

[226] Wang M., Wang W., Ma B., Shen W., Liu L., Cao K., Chen S., Huang W. (2021). Lead-Free Perovskite Materials for Solar Cells. Nano-Micro Lett..

[227] Gong J., Darling S.B., You F. (2015). Perovskite photovoltaics: Life-cycle assessment of energy and environmental impacts. Energy Environ. Sci..

[228] Matteocci F., Cinà L., Di Giacomo F., Razza S., Palma A.L., Guidobaldi A., D’Epifanio A., Licoccia S., Brown T.M., Reale A. (2016). High efficiency photovoltaic module based on mesoscopic organometal halide perovskite. Prog. Photovolt. Res. Appl..

[229] Razza S., Di Giacomo F., Matteocci F., Cinà L., Palma A.L., Casaluci S., Cameron P., D’Epifanio A., Licoccia S., Reale A. (2015). Perovskite solar cells and large area modules (100 cm^2^) based on an air flow-assisted PbI*2* blade coating deposition process. J. Power Sources.

[230] Lang F., Gluba M.A., Albrecht S., Rappich J., Korte L., Rech B., Nickel N.H. (2015). Perovskite solar cells with large-area CVD-graphene for tandem solar cells. J. Phys. Chem. Lett..

[231] Mo Y., Shi J., Zhou P., Li S., Bu T., Cheng Y.-B., Huang F. (2019). Efficient Planar Perovskite Solar Cells via a Sputtered Cathode. Sol. RRL.

[232] Yang P., Wang J., Zhao X., Wang J., Hu Z., Huang Q., Yang L. (2019). Magnetron-sputtered nickel oxide films as hole transport layer for planar heterojunction perovskite solar cells. Appl. Phys. A Mater. Sci. Process..

[233] Fagiolari L., Bella F. (2019). Carbon-based materials for stable, cheaper and large-scale processable perovskite solar cells. Energy Environ. Sci..

[234] Bella F., Griffini G., Correa-Baena J.P., Saracco G., Grätzel M., Hagfeldt A., Turri S., Gerbaldi C. (2016). Improving efficiency and stability of perovskite solar cells with photocurable fluoropolymers. Science.

[235] Charles U.A., Ibrahim M.A., Teridi M.A.M. (2018). Electrodeposition of organic–inorganic tri-halide perovskites solar cell. J. Power Sources.

[236] Dharmadasa I.M., Rahaq Y., Alam A.E. (2019). Perovskite solar cells: Short lifetime and hysteresis behaviour of current–voltage characteristics. J. Mater. Sci. Mater. Electron..

[237] Raifuku I., Ishikawa Y., Bourgeteau T., Bonnassieux Y., Roca P.I.C., Uraoka Y. (2017). Fabrication of perovskite solar cells using sputter-processed CH_3_NH_3_PbI_3_ films. Appl. Phys. Express.

[238] Meng L., You J., Yang Y. (2018). Addressing the stability issue of perovskite solar cells for commercial applications. Nat. Commun..

[239] Holzhey P., Saliba M. (2018). A full overview of international standards assessing the long-term stability of perovskite solar cells. J. Mater. Chem. A.

[240] Saliba M., Stolterfoht M., Wolff C.M., Neher D., Abate A. (2018). Measuring Aging Stability of Perovskite Solar Cells. Joule.

[241] Uddin A., Upama M.B., Yi H., Duan L. (2019). Encapsulation of Organic and Perovskite Solar Cells: A Review. Coatings.

[242] Wohlgemuth J., Kurtz S. Photovoltaic Module Qualification Plus Testing. Proceedings of the 2014 IEEE 40th Photovoltaic Specialist Conference, PVSC 2014.

[243] Corsini F., Griffini G. (2020). Recent progress in encapsulation strategies to enhance the stability of organometal halide perovskite solar cells. J. Phys. Energy.

[244] Rostan N.F., Sepeai S., Mohamad Yunus R., Ahmad Ludin N., Mat Teridi M.A., Ibrahim M.A., Sopian K. (2020). Optoelectronic and morphology properties of perovskite/silicon interface layer for tandem solar cell application. Surf. Interface Anal..

[245] Microquanta Achieves 14.24% Efficiency with Large-Area Perovskite Solar Module—Pv Magazine International. https://www.pv-magazine.com/2019/10/24/microquanta-achieves-14-24-efficiency-with-large-area-perovskite-solar-module/.

[246] Microquanta Announces 14.24% Efficiency with Large-Area Perovskite Solar Module|Perovskite-Info. https://www.perovskite-info.com/microquanta-announces-1424-efficiency-large-area-perovskite-solar-module.

[247] Zheng J., Lau C.F.J., Mehrvarz H., Ma F.J., Jiang Y., Deng X., Soeriyadi A., Kim J., Zhang M., Hu L. (2018). Large area efficient interface layer free monolithic perovskite/homo-junction-silicon tandem solar cell with over 20% efficiency. Energy Environ. Sci..

[248] Ho-Baillie A. (2018). Perovskites cover silicon textures. Nat. Mater..

[249] Fatima N., Karimov K.S., Elsmani M.I., Ibrahim M.A. (2021). Photodetector based on silicon-graphene heterojunction fabricated through rubbing-in technology. Optik (Stuttgart).

[250] Zheng J., Mehrvarz H., Liao C., Bing J., Cui X., Li Y., Goncąles V.R., Lau C.F.J., Lee D.S., Li Y. (2019). Large-area 23%-efficient monolithic perovskite/homojunction-silicon tandem solar cell with enhanced uv stability using down-shifting material. ACS Energy Lett..

